# Digital Workflow for the Restoration of the Vertical Dimension of Occlusion Evaluated by 3D Stereophotogrammetry

**DOI:** 10.1111/jerd.70030

**Published:** 2025-09-15

**Authors:** Ernesto B. Benalcazar‐Jalkh, Laura F. de Carvalho, Abbas Zahoui, Maria Giulia R. Pucciarelli, Simone Soares, Marcos Celestrino, Estevam A. Bonfante

**Affiliations:** ^1^ Department of Prosthodontics and Periodontology Bauru School of Dentistry, University of Sao Paulo Bauru SP Brazil; ^2^ Hospital for Rehabilitation of Craniofacial Anomalies University of São Paulo São Paulo SP Brazil; ^3^ Laboratório Aliança São Paulo SP Brazil

**Keywords:** 3D imaging, digital workflow, full‐mouth rehabilitation, stereophotogrammetry, vertical dimension of occlusion

## Abstract

**Objective:**

To present a digital workflow approach for the full‐mouth rehabilitation of severely worn dentition and to evaluate the impact of vertical dimension of occlusion (VDO) rehabilitation on facial parameters assessed by 3D stereophotogrammetry throughout all treatment steps.

**Clinical Considerations:**

Extensive tooth wear frequently demands the determination of a new VDO for the prosthodontic treatment. While digital tools have been used to establish hybrid or fully digital workflows in prosthodontics, a validation of accuracy between the planification stage and final VDO, as well as resulting linear and volumetric changes in the soft tissues after treatment, has not been reported utilizing tridimensional facial reconstruction systems. The clinical report describes the case of a 34‐year‐old patient with generalized erosive tooth wear and severe tooth sensitivity. After diagnosis, the patient was treated with a minimally invasive digitally driven full‐mouth rehabilitation using lithium–disilicate bonded restorations, and stereophotogrammetry was used for the evaluation of facial changes before, at the planning stage, and after VDO rehabilitation.

**Conclusions:**

The fully digital workflow allowed for a predictable rehabilitation of the VDO, where similar stereophotogrammetric measurements were observed during the mock‐up and at the end of the treatment. 3D stereophotogrammetry imaging allowed to quantitatively assess differences in facial appearance after VDO rehabilitation.

**Clinical Significance:**

The conscious application of fully digital workflows enables predictable achievement of both functional and esthetic parameters in the rehabilitation of VDO. 3D stereophotogrammetry confirmed similar facial measurements between the initial condition, mock‐up stage, and treatment outcome.

## 1 Introduction

Oral rehabilitation procedures are increasingly sought by patients, seeking comfort, esthetics, and function. Therapeutic possibilities vary from the most straightforward and simple treatments with the replacement of a single element to more complex cases involving complete oral rehabilitations supported by tooth or osseointegrated implants. Prosthodontists frequently face the challenge of reestablishing esthetics and function in patients with severe tooth wear. In such scenarios, most of the occlusal references for reconstruction are missing, and the determination of new maxillomandibular relationships is needed. Furthermore, severe tooth wear may be accompanied by facial esthetic alterations that require careful examination of the patient's occlusal parameters, especially regarding the vertical dimension of occlusion (VDO) [1, 2].

VDO is defined as the distance between two selected anatomic or marked points (usually one on the tip of the nose and the other on the chin) when in the maximal intercuspal position [3]. The decrease in VDO is often associated with the presence of parafunctional habits, extensive tooth wear, and/or loss of posterior occlusal stability [4, 5]. The evaluation of VDO is traditionally associated with the assessment of the vertical dimension of rest (VDR), defined as the postural jaw relation when the patient is resting comfortably in an upright position and the condyles are in a neutral, unstrained position [3]. Both dimensions may change with age as well as with loss of mineralized tooth structure [6], and their evaluation, determination, and importance have been the center of debates in the field of dental prosthodontics.

The therapeutic alteration of a patient's VDO is not an exact science and has been better described as a clinical treatment based on the evaluation of facial parameters and esthetic references [1]. Among them, the understanding of the interocclusal distance (IOD), defined as the difference between the VDR and the VDO [3] has been considered pivotal for a successful determination of the patient's VDO [7]. Classical literature has reported a wide clinical variation of the IOD, ranging from 1 to 9.5 mm. However, 3–4 mm has been considered a median IOD in normal dentulous individuals and frequently used as an important parameter for VDO rehabilitation [1]. Even severely worn dentition may still present these average distances preserved, and potential changes in the existing VDO may take place for restorative convenience [1, 8, 9, 10].

Traditional methods to determine de VDO include esthetic [11, 12], metric [13, 14], physiologic [15] phonetic [16, 17], and cephalometric [18, 19] assessments. Intriguingly, while the establishment of a VDO for patients is a vital step to achieve pleasing esthetics and harmonious function, there is no singular VDO determination method that is better than another, nor a completely accurate method for determining it [4]. This lack of consensus within the literature has led clinicians to assess VDO using clinical judgment based on subjective comparisons and combining different techniques [20, 21, 22, 23, 24, 25]. In the early 1900s, changes in VDO were classically correlated with diverse symptoms, such as muscle pain, temporomandibular joint discomfort, headaches, as well as tooth grinding and clenching [26, 27]. However, most of these assumptions were derived from personal opinions and case reports that not only lacked evidence but also remained widespread for a long time. Currently, a critical evaluation of clinical studies suggests that the stomatognathic system has a great ability to adapt to increases in VDO [28, 29]. Although augmentation of VDO in up to 5 mm has been reported to be safe, some patients may present mild transient symptoms, mostly self‐limited, with a tendency to resolve within 2 weeks [29, 30, 31].

Along with technological developments, the application of fully digital workflows is increasingly implemented by dentists and lab technicians to allow for predictable and time‐efficient reconstructive treatments. Intraoral scanners, 3D printers, planning software, and CAD‐CAM systems are widely used in dental clinics. However, the management of complex cases involving changes in the VDO might be challenging if analog traditional procedures for oral rehabilitation are not executed to feed information into the digital workflow process. Regardless of technological advances, the fundamental concepts of oral rehabilitation remain the key point to achieve long‐lasting functional and esthetic outcomes. Despite the fact that consensus in desired occlusal schemes may not always be achieved as well as in definitions of static positions such as centric relation (CR) [32, 33, 34], the prosthodontist will have to resolve the challenge of recording an accurate maxillo‐mandibular relationship to be transferred to the dental laboratory at some stage during the treatment [35]. Moreover, the transfer of maxillo‐mandibular 3D positioning to lab technicians requires the deliberate application of digital tools along with clinical strategies to ensure predictable outcomes from planning to mock‐up or provisional to final prostheses installation.

Alongside clinical parameters, the material selection, thickness, and tooth preparation procedures have a significant impact on the clinical outcome of oral rehabilitations. Conservative approaches that prioritize tooth structure preservation have been reported to enhance bonding resistance, maintain tooth strength, and reduce the risk for post‐operative sensibility [36]. Among the available options, lithium disilicate reinforced glass–ceramics have gained popularity due to their well‐established bonding protocols and outstanding performances reported in long‐term clinical trials [37, 38]. However, diseases such as gastroesophageal reflux disease (GERD) have been strongly associated with dental erosion [39], and recognized as an important risk factor for sleep bruxism [40]. Such chemical–mechanical association synergistically promotes tooth wear and, depending on its severity, may require restorative interventions [40]. Therefore, in such cases of severe tooth wear caused by GERD and sleep bruxism, minimally invasive dentistry involves being sufficiently invasive to create room for the restorative material.

Moreover, the management of complex cases that involve changes in the maxillomandibular positions has a significant impact on the facial soft tissue's support. Among the methods available for quantitative facial analysis, stereophotogrammetry, also known as three‐dimensional photography (3D photograph), has stood out as a gold standard in the 3D evaluation of facial soft tissues. Through specialized software, 3D images overlapping can be used to detect facial modifications (linear, angular, surface, perimeter and volume) during the prosthodontic planning, mock‐up or provisional stage, and final treatment [41, 42, 43, 44].

Although facial changes produced by increasing VDO are frequently reported through conventional 2D photography before and after prosthodontic treatments [45, 46, 47], the evaluation of accuracy between the planification stage and the outcome of VDO rehabilitation with quantitative analyses of changes in the facial soft tissues has been scarcely reported utilizing tridimensional reconstruction systems. Therefore, the objective of this article is to present a digital workflow approach for the full‐mouth rehabilitation of severely worn dentition and to evaluate through 3D stereophotogrammetry the effect of VDO rehabilitation in facial parameters at baseline, planned mock‐up, and final treatment.

## 2 Materials and Methods

**FIGURE 1:**
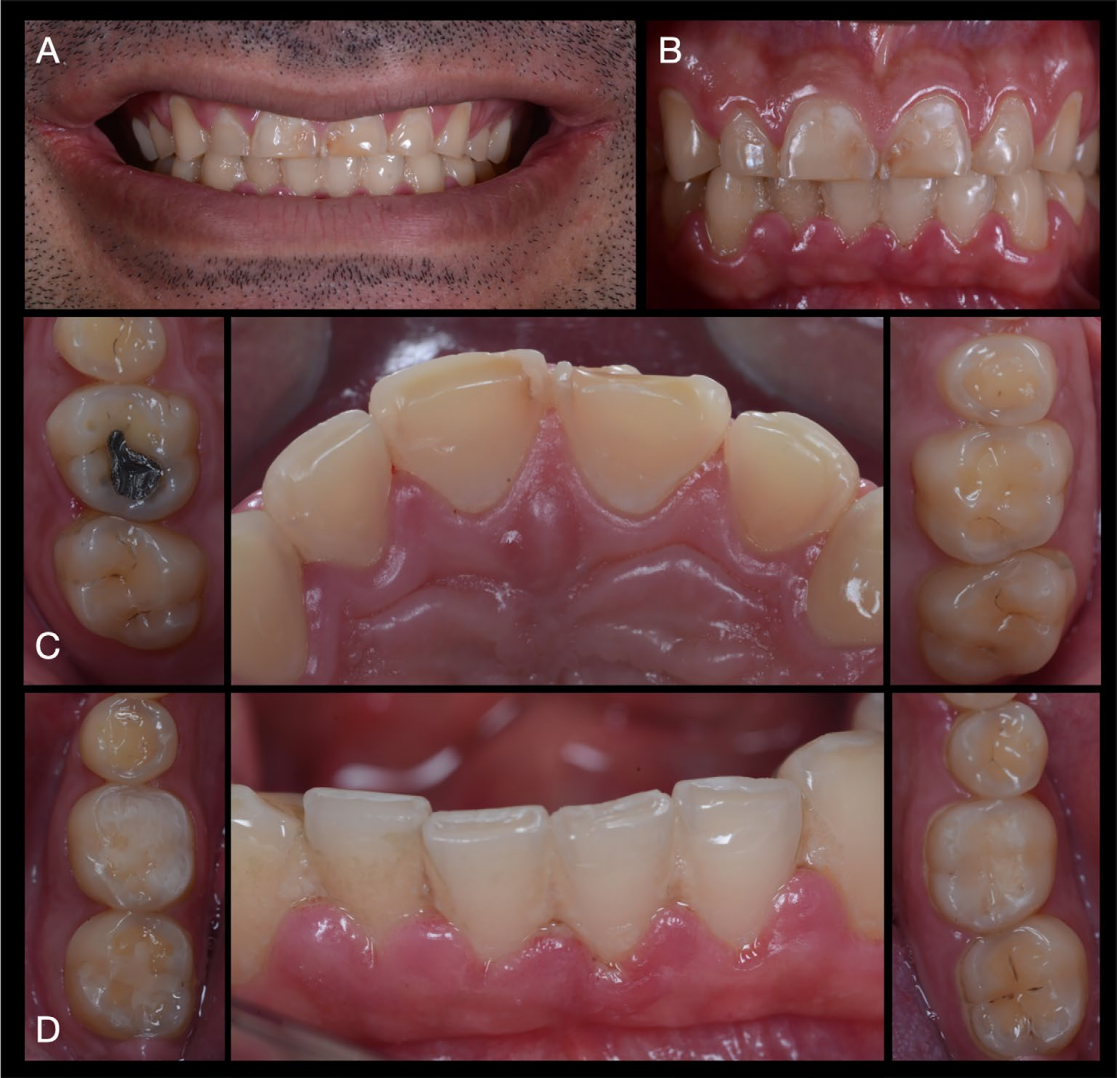
Initial condition of the patient presented in close‐up (A), and intraoral (B) frontal views. Occlusal, palatal, and anterior views of the maxillary (C) and mandibular (D) arches are also presented to depict the pathognomonic erosive generalized lesions and gingival inflammation.

A 27‐year‐old male patient first visited the clinic of prosthodontics at the University of São Paulo—Bauru School of Dentistry, with severe dental sensitivity as well as complaints about esthetics and function. In the anamnesis, the patient self‐reported severe gastroesophageal reflux and sleep bruxism. Moreover, no other systemic disorders or relevant medical history were reported by the patient. At close‐up and intraoral examination (Figure 1A,B, respectively), significant wear of dental structure was observed, as well as plaque deposition, gingival inflammation, and unsatisfactory direct resin‐composite restorations. Additionally, the patient reported that the severe tooth sensitivity impeded him from performing proper dental hygiene, which promoted gingival inflammation. Figure 1C,D present the intraoral condition of the patient, with pathognomonic evidence of erosive lesions in the palatal surface of maxillary incisors and occlusal surfaces of posterior teeth.

Typical signs of erosive tooth wear were observed on occlusal maxillary and mandibular surfaces with cupping of the cusps and flattening of the occlusal structures. Moreover, flattening of axial surfaces and the presence of wide concavities in the palatal surface of maxillary incisors confirmed the diagnosis of erosive wear of dental structures [48]. Therefore, the basic erosive wear examination (BEWE) scoring system was used to assess the patient's risk level [49]. With a cumulative score of 10, the patient was considered at middle risk level, and the management consisted of developing strategies to eliminate etiological factors for tissue loss. In the first place, due to active acid reflux and concerns regarding the potential development of medical conditions related to the disease, such as laryngeal cancer [50, 51, 52], the patient was referred for gastroenterologist treatment, and a conservative treatment and monitoring of erosive wear was planned for his dental condition. However, the patient did not adhere to the proposed dental treatment at that time and pursued treatment at a private practice elsewhere.

**FIGURE 2:**
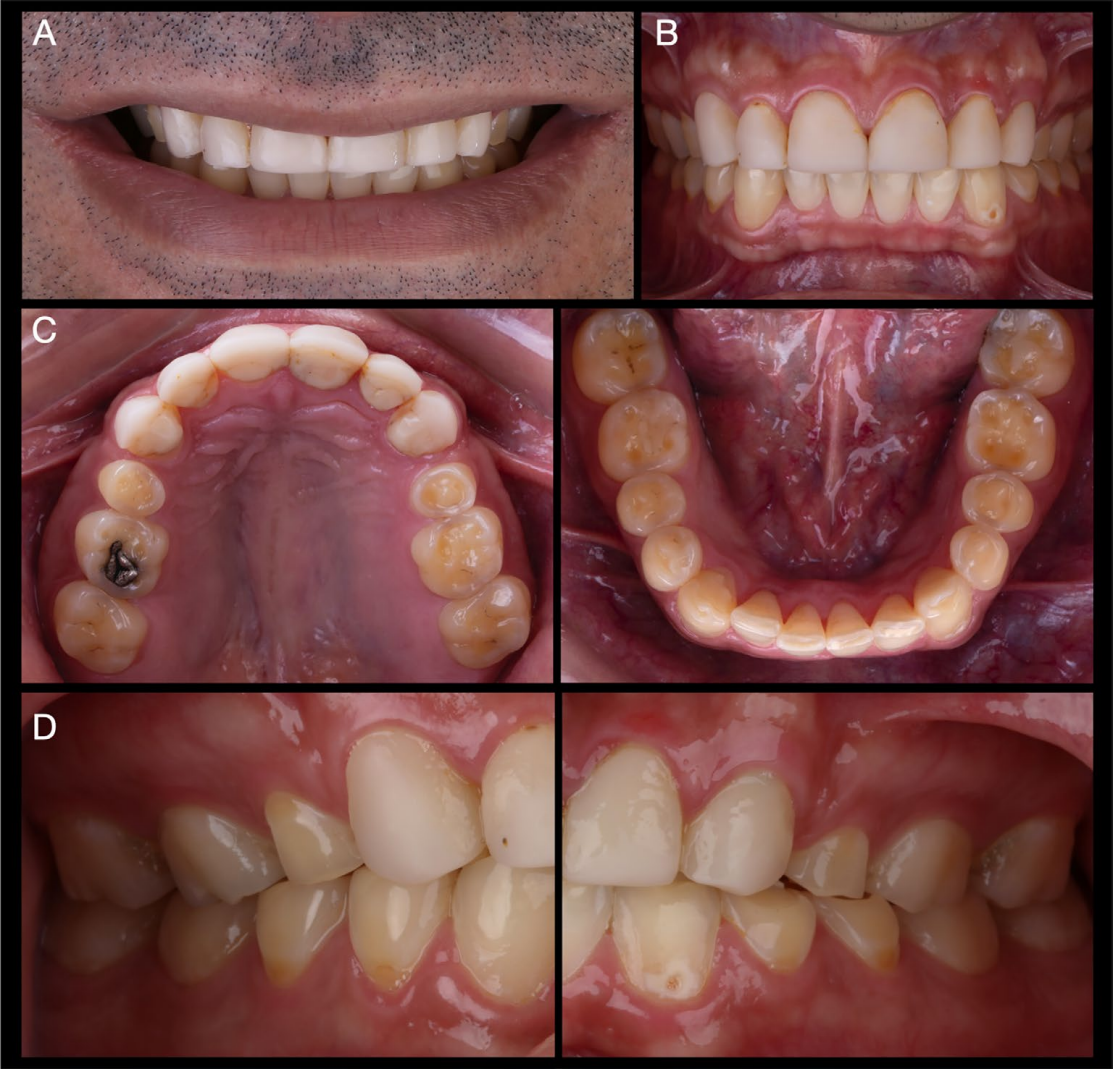
Condition of the patient 7 years after the first appointment. Resin composite buccal and palatal veneers were performed in a private practice 3 years prior to the return of the patient. Frontal close‐up (A) and intraoral (B) views. Occlusal (C) and lateral (D) views depict a significant progression of erosive lesions, particularly in the occlusal surfaces of maxillary and mandibular molars and pre‐molars, where a significant amount of dentin exposure was observed, and the patient reported severe dental sensitivity.

Seven years after the first appointment, at 34 years old, the patient attended the clinic looking for a comprehensive dental treatment. The patient reported a restorative treatment in the anterior region at a private practice 2 years prior to this appointment. At close‐up and intraoral examination (Figure 2) the treatment with resin composites presented cervical over‐contours, wear, and marginal pigmentation. Moreover, the patient's chief complaint remained the same: severe tooth sensitivity. The intraoral examination of posterior regions depicted a significant progression of erosive lesions regarding the condition observed 7 years before, where occlusal resin composites were completely lost due to the association of erosive wear and self‐reported sleep bruxism confirmed by his partner. Moreover, it was possible to observe that the previous restorative treatment included the palatal surface of maxillary incisors with over‐contoured and pigmented restorations. At BEWE, a cumulative score of 15 was recorded. This denoted a severe progression of erosive tooth wear and a high‐risk level, which demanded special care. The patient was referred once again to a gastroenterologist.

**FIGURE 3:**
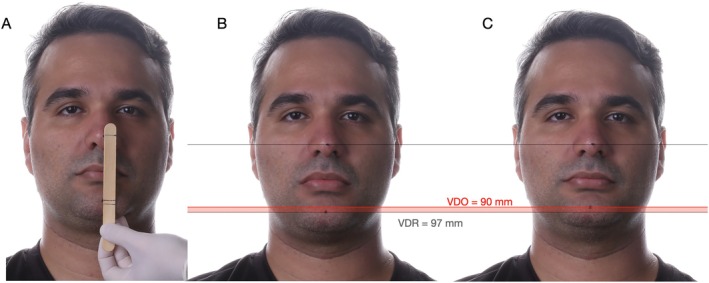
Vertical dimension of occlusion (VDO) and vertical dimension of rest (VDR) evaluation through the metric method utilizing an examination stick and two arbitrary marks, one in the nose and another one in the chin (A). At evaluation, a difference between VDR (B) and VDO (C) provided an interocclusal distance at rest of 7 mm.

A first step for the assessment of VDO during extraoral evaluation was performed through the metric method, where the distance between a marked point on the tip of the nose and another on the chin was used to determine the VDO, VDR, and the IOD, as presented in Figure 3. Metric evaluation depicted an IOD of 7 mm, and the esthetic evaluation demonstrated characteristic signs of diminished VDO, suggesting that increasing the VDO would be possible and convenient for the oral rehabilitation of the patient. At this point, 3D stereophotogrammetric evaluation of the initial condition was performed to assess the VDO at baseline and will be detailed in Section 2.5.

### 2.1 Determination of VDO and Maxillo‐Mandibular Parameters

**FIGURE 4:**
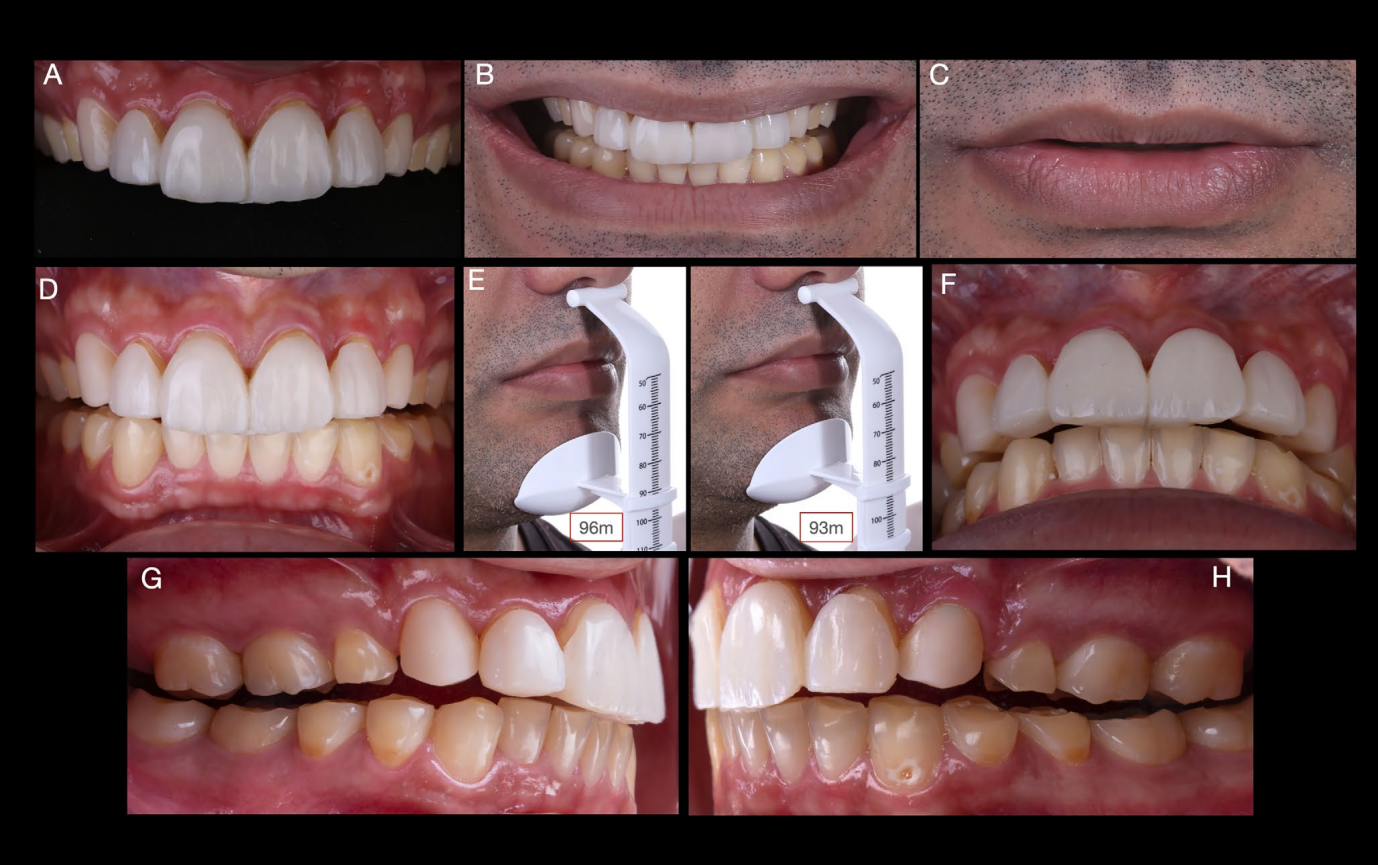
Determination of the vertical dimension of occlusion utilizing an esthetic jig manufactured with resin composite IPS Empress Direct (A1, Ivoclar Vivadent, Liechtenstein) (A). The removable device is manufactured over the patient's teeth and allows for an initial determination of incisor's height (B, C), vertical dimension of occlusion (D, E), centric relation (F), and the space for the vertical dimension of occlusion augmentation (G, H).

To determine the VDO, a modified jig to also serve as an esthetic reference was manufactured in resin composite (Figure 4A). The aim of this esthetic jig, produced with acrylic or composite resin, is 3‐fold, allowing the determination of: (1) Central incisor's tentative height to achieve an esthetic position of the incisal edge with the lip at rest and during smile. As depicted in Figure 4B,C, the incisal edge was placed at the level of the lip at rest and a harmonic distribution was corroborated during smile. This step seeks to remove from the dental technician the responsibility of arbitrarily determining the height of the central incisors based on average dimensions (e.g., 10 mm) which may lead to inconsistencies and lack of customization [53]; (2) VDO, which is obtained through the association of metric, and esthetic methods with the jig in position and through clinical adjustments to achieve an IOD of approximately 3 mm, as presented in Figure 4C; and (3) CR, determined by the absence of posterior contacts with a single contact in the anterior region (Figure 4D) and physiological unforced mandibular manipulation. The space for VDO augmentation is depicted in lateral views presented in Figure 4E,F.

### 2.2 Digital Workflow for Planning Complete Oral Rehabilitation

**FIGURE 5:**
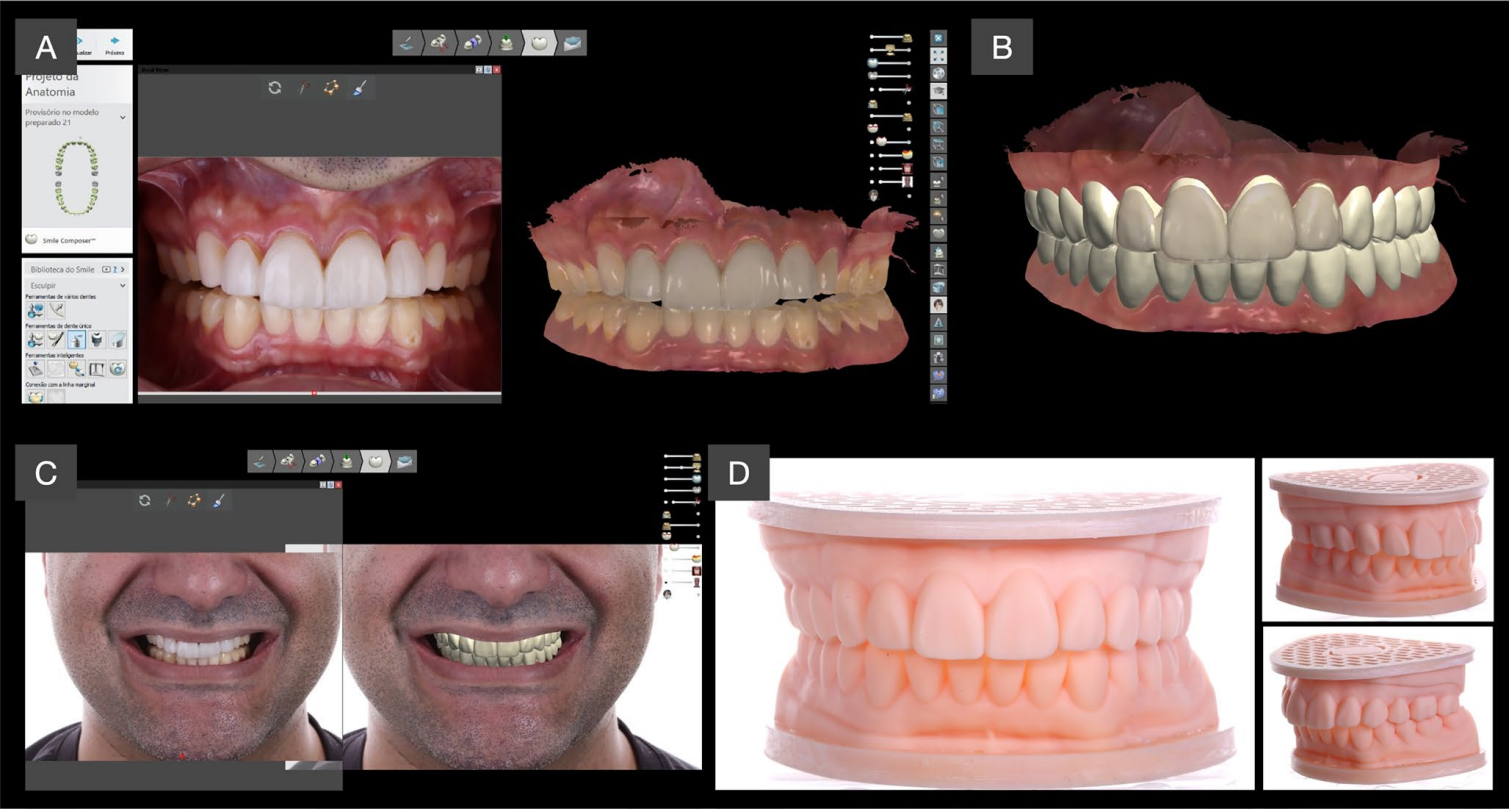
Three‐dimensional virtual models were imported to planning software (A) and a full‐mouth digital teeth arrangement (B) was performed based on the esthetic maxillo‐mandibular references provided by the esthetic jig and extra‐oral photographs, respectively. The virtual planning was 3D printed, and physical models were obtained (D).

To establish a fully digital workflow for oral rehabilitation, the patient's arches were intraorally scanned (TRIOS 3, 3Shape) with and without the jig, and the maxillomandibular relation was recorded with the esthetic jig in place to transfer esthetic and functional parameters (Figure 5A). Additionally, an extraoral photographic protocol was performed to provide facial parameters for three‐dimensional planning. The scans and collected information were then used by the lab technician (M.C.) to create a virtual design of the future rehabilitation, as presented in Figure 5. The virtual planning was then 3D‐printed to obtain physical models that were used to obtain silicone matrices of the maxillary and mandibular arches.

**FIGURE 6:**
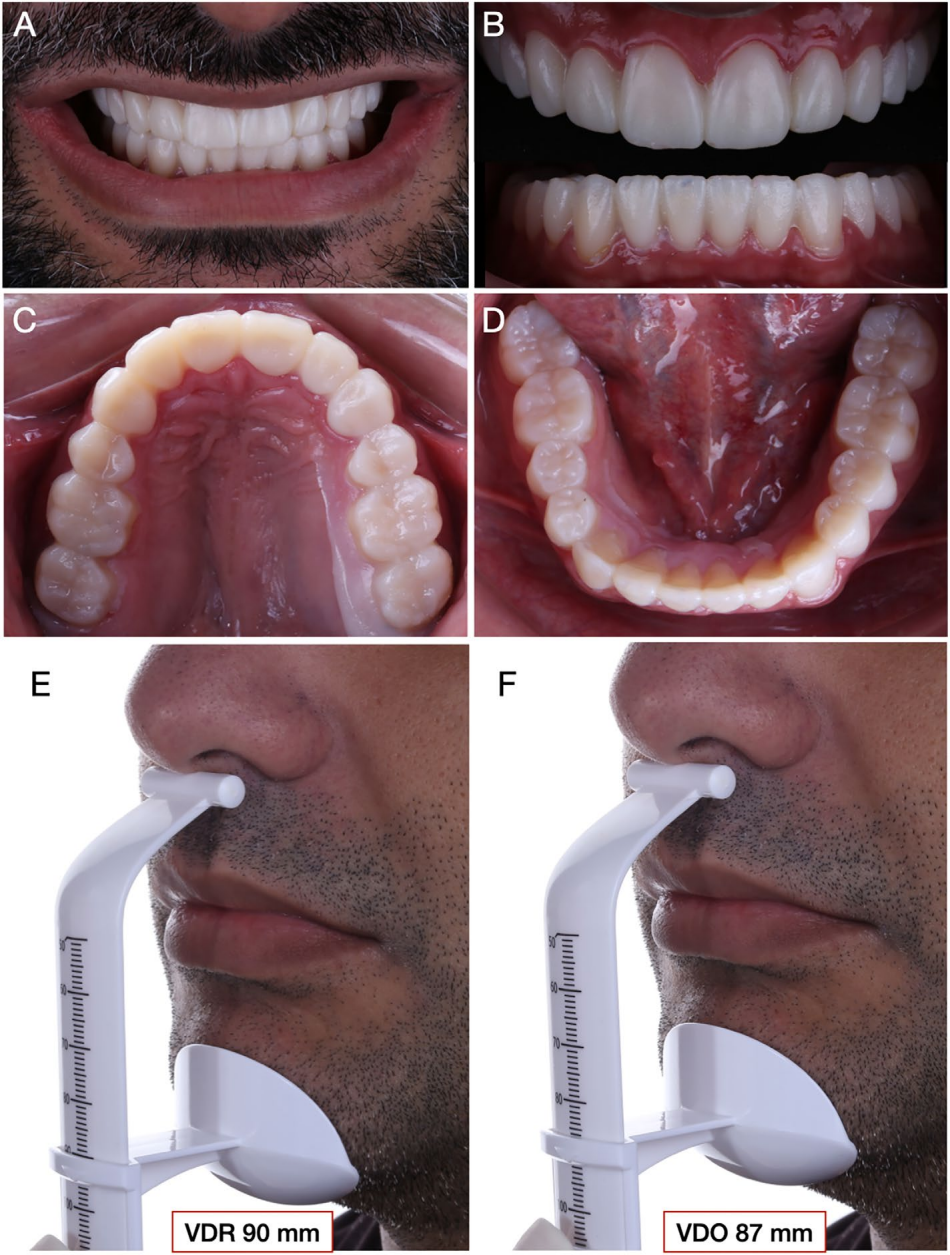
Full mouth mock‐up performed with bisacrylic resin (Structure 2, A1, Voco, Germany) for esthetic and functional evaluations. (A) Close‐up view of the patient's smile with the mock‐up in position. (B) Frontal and occlusal (C, D) views of maxillary and mandibular arches. (E, F) Evaluation of the vertical dimension of rest and vertical dimension of occlusion with the mock‐up in place revealed an IOD of approximately 3 mm, similar to that observed with the esthetic jig in place.

Full arch maxillary and mandibular mock‐up (Figure 6) was performed with resin (Structure 2, A1, Voco, Germany) after isolation of pre‐existing restorations with glycerin to facilitate mock‐up removal. Subsequently, VDO, CR, occlusion, esthetic, and phonetic parameters were evaluated to assure the proper establishment of all maxillo‐mandibular relations. At VDO evaluation with the mock‐up, an IOD of approximately 3 mm was observed, similar to that previously determined using the esthetic jig. At this point, pictures for the second 3D stereophotogrammetric evaluation were obtained with the mock‐up in place. The methods and results will be presented and discussed in Section 2.5.

After the confirmation of occlusal and esthetic parameters, the diagnostic and planning stages were successfully completed, and the treatment phase was set to start with a clear and predictable desired outcome.

The treatment plan consisted of minimally invasive digitally driven full‐mouth rehabilitation using full (maxillary incisors and canines) and partial lithium–disilicate crowns (maxillary and mandibular molars and premolars), and laminate veneers (mandibular incisors and canines). An occlusal splint was also planned to limit deleterious effects of sleep bruxism. Additionally, stereophotogrammetry was used for the evaluation of facial changes at baseline, the planning phase with the bisacrylic mock‐up, and after VDO rehabilitation.

### 2.3 Preparation Sequence and Digital Record of Maxillo‐Mandibular Relationship

**FIGURE 7:**
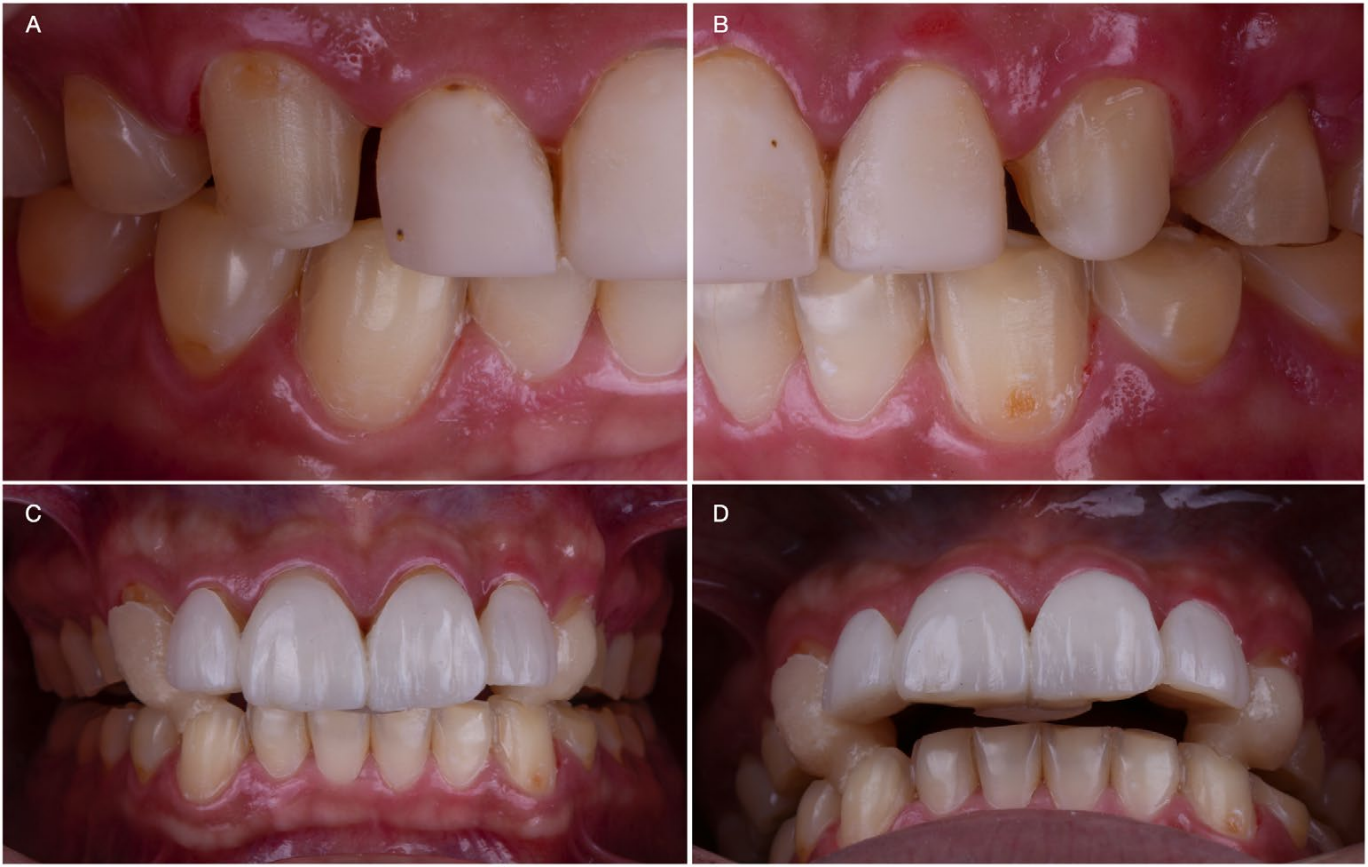
Preparation sequence started with full crown preparation of maxillary canines and veneer preparation of mandibular canines (A, B). Then, registration copings were manufactured over the maxillary canines with acrylic resin, and registration was performed with the esthetic jig in position to transfer the recorded maxillo‐mandibular relationships approved in the mock‐up, such as CR and VDO (C, D).

To establish a minimally invasive approach, erosive lesions were restored with a glass‐ionomer material (Gold label, GC Corporation, Japan). Before tooth preparation, it is paramount to consider that the complete set of parameters utilized as guidance for planning is based on the esthetic jig manufactured during planning. Therefore, a preparation sequence that allows for the preservation and transfer of the registered maxilla‐mandibular information should be prioritized. In the present case, the esthetic jig was supported by the maxillary incisors and occluded against the mandibular incisors. Therefore, the first step comprised the preparation of the maxillary and mandibular canines for full crowns and laminate veneers, respectively (Figure 7A,B). This allowed for the manufacture of a registration coping over the maxillary canines with a stable contact against the mandibular canines, which secured the registration of the occlusal parameters determined by the esthetic jig as depicted in Figure 7C,D.

**FIGURE 8:**
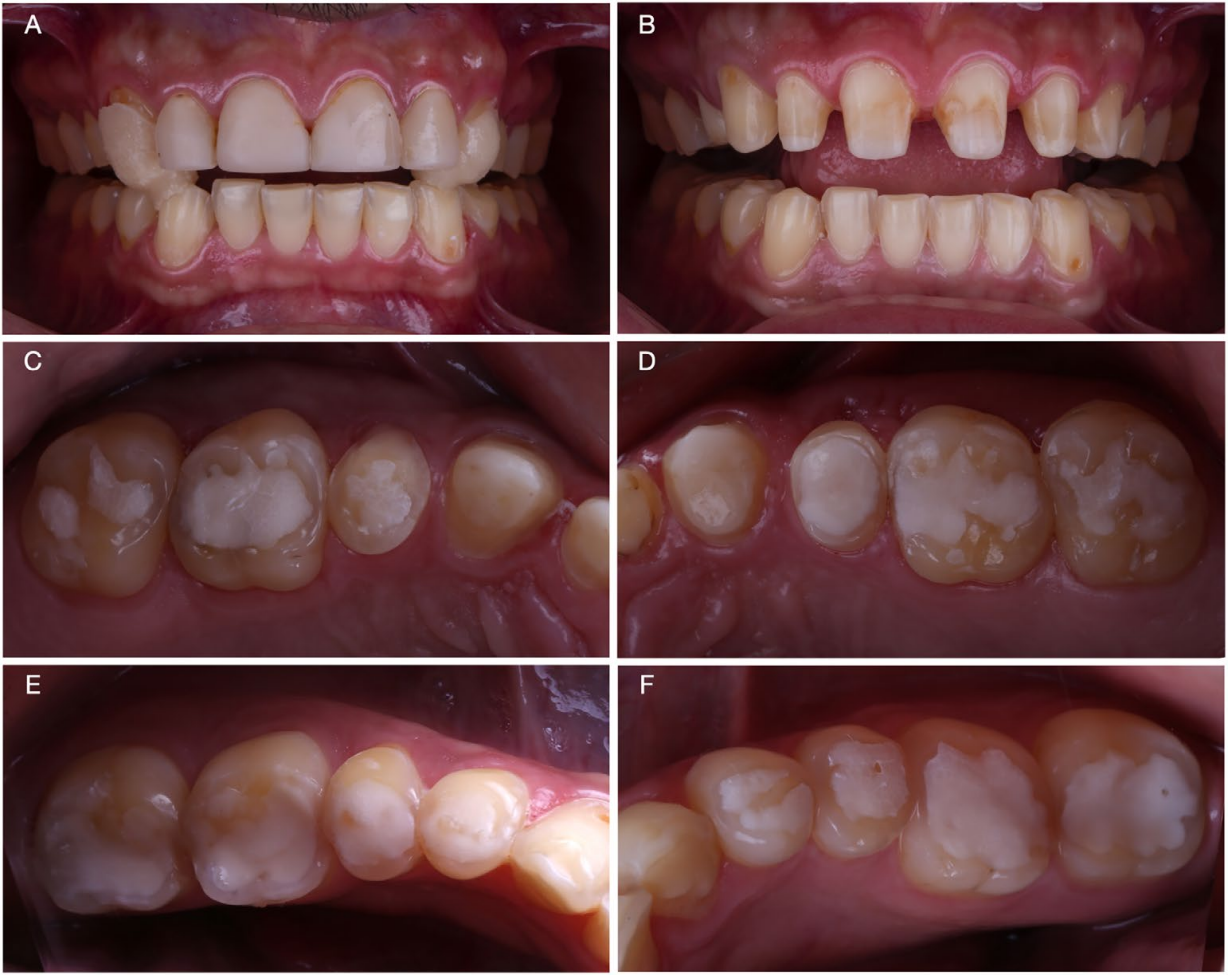
(A) Frontal view of registration copings manufactured over the maxillary canines with stable contact against the prepared mandibular canines. (B–F) Full mouth preparations for complete, partial crowns and veneers, as per the treatment plan.

Once the stability of the registration copings was verified (Figure 8A), full mouth tooth preparation was performed according to the treatment plan (Figure 8B–F). Particular attention was given to provide sufficient thickness to hide pigmented substrates in the anterior region (approximately 1 mm of thickness) and minimal occlusal thickness in the posterior region by performing a mock‐up‐oriented preparation. Occlusal preparation was performed to provide 1.5 mm of thickness at cusps and 0.5–1 mm of thickness in the fossa. Reduced occlusal thickness was adopted due to the limited substrate height in the molar region regarding the IOD achieved during planning.

**FIGURE 9:**
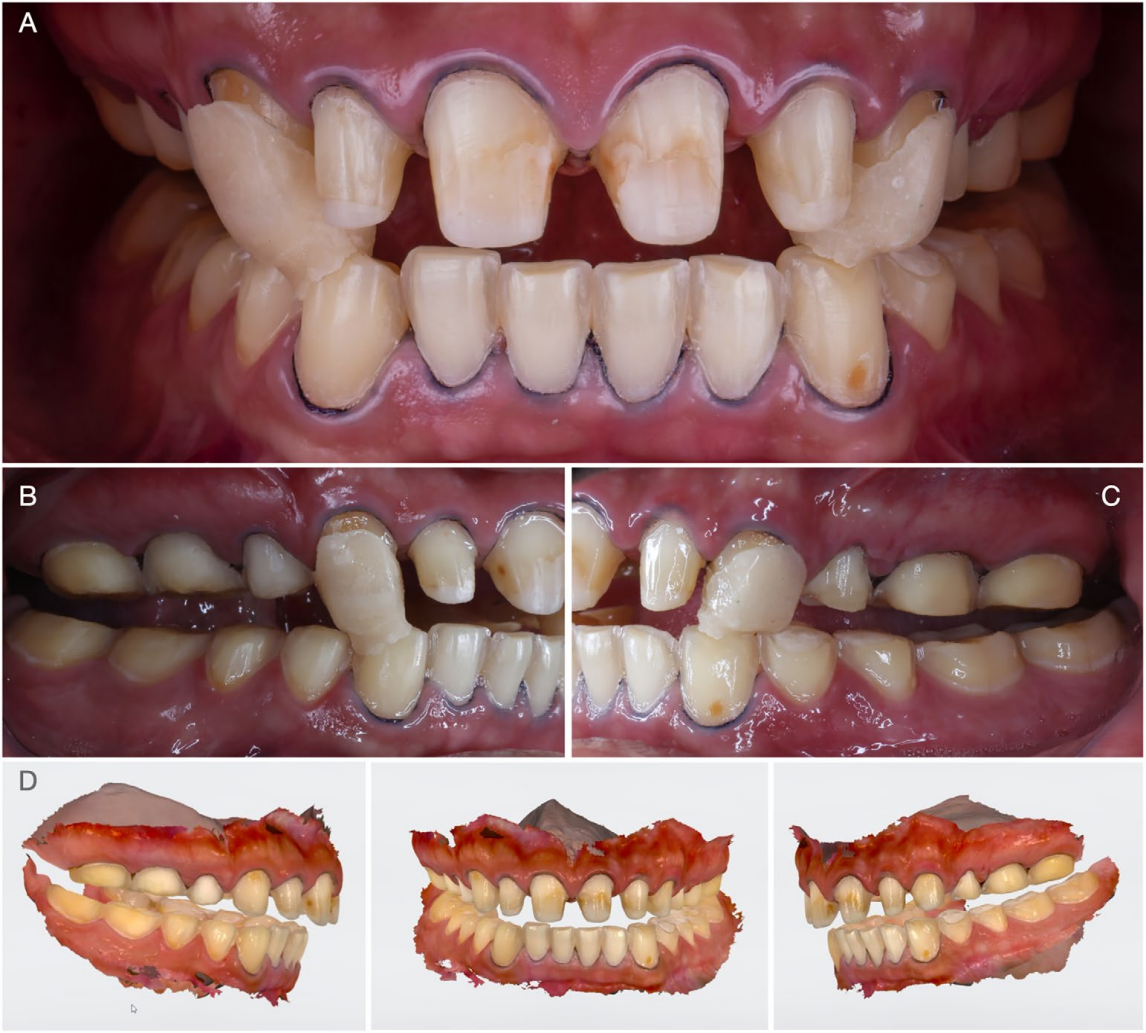
(A) Frontal view of prepared maxillary and mandibular arches with #000 cord and registrations copings in position. (B–C) Lateral views of maxillo‐mandibular relationships to be intraorally scanned. (D) Digital models obtained with intraoral scanning of both arches and registration with registration copings in position.

After finalizing dental preparations, digital scanning was performed using the double cord technique. During scanning, the thinner #000 cord (Ultrapak, Ultradent Products, USA) was kept inside the gingival sulcus while the thicker #00 was removed. To register the maxillo‐mandibular relation, the registration step utilizing the intraoral scanner was performed with the registration copings placed over the canines and in stable contact against the mandibular canines, which provided a stable and reproducible relation depicted in Figure 9A–C. The files obtained from intraoral scanning and the occlusal records were sent to the dental laboratory to manufacture lithium disilicate restorations (Figure 9D).

**FIGURE 10:**
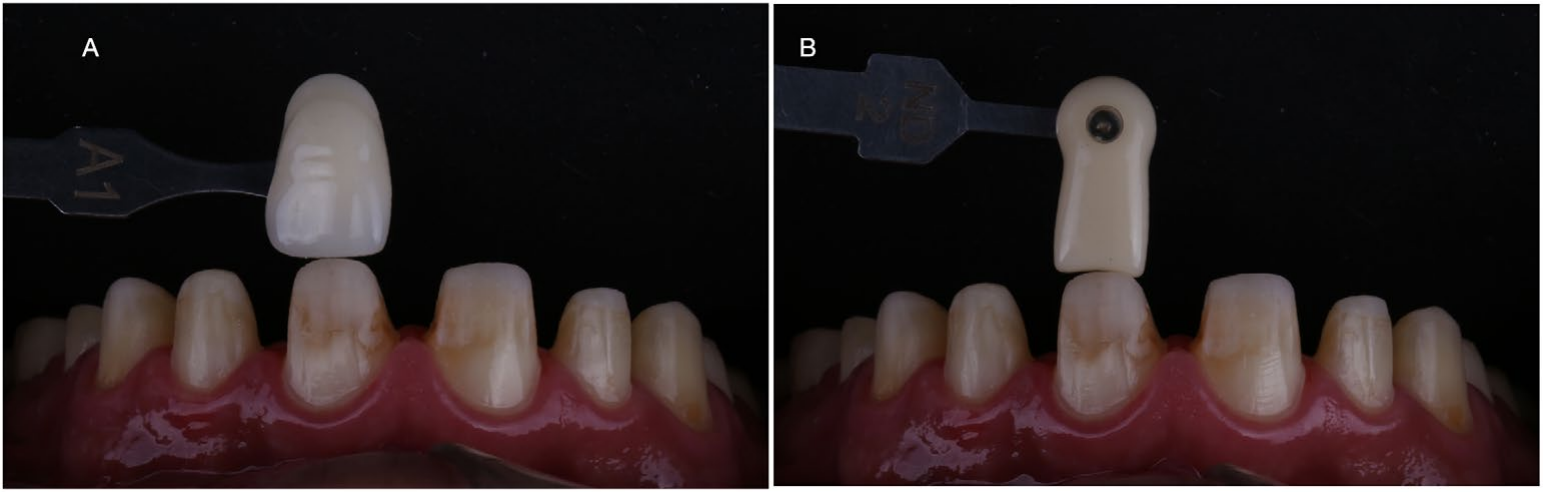
(A) Selection of the final color of the lithium disilicate restorations. (B) Color selection of the substrate to efficient communication with dental technician.

Substrate color was registered with the aid of dental photography as well as its relationship with the desired color for the final restorations (Figure 10A,B). The prepared teeth were then provisionally restored with bisacrylic resin (Tempsmart A1 GC Corporation, Japan) utilizing the matrices used for mockup evaluation.

### 2.4 Lithium–Disilicate Bonded Restorations

**FIGURE 11:**
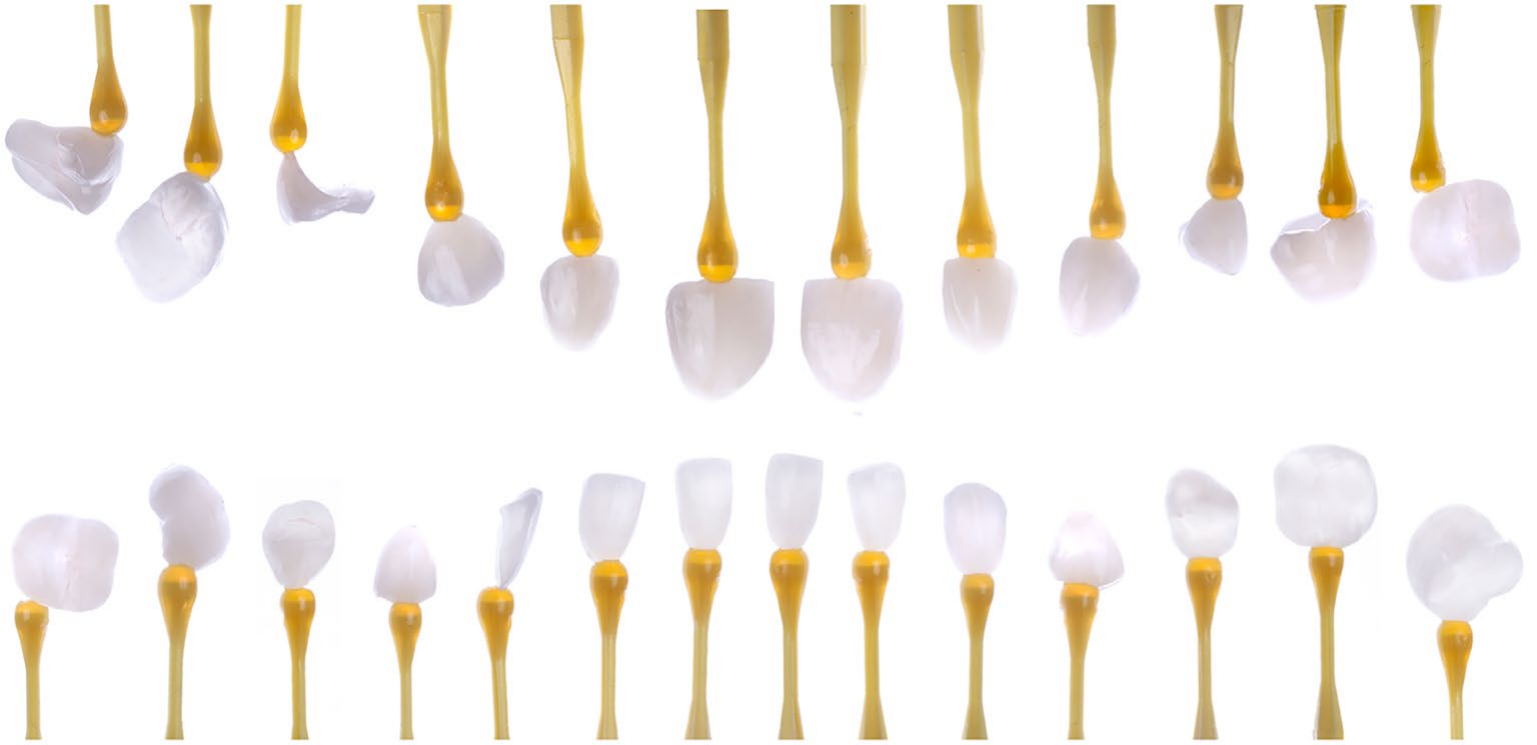
Pressed e.max lithium–disilicate restorations produced by M.C. (Laboratório Aliança, SP).

Twenty‐six IPS e.max Press (Ivoclar Vivadent, Liechtenstein) restorations were produced by wax milling followed by the lost‐wax technique and injection of ingots HT, shade A1. Finally, monolithic restorations were stained to achieve the esthetic outcomes presented in Figure 11.

**FIGURE 12:**
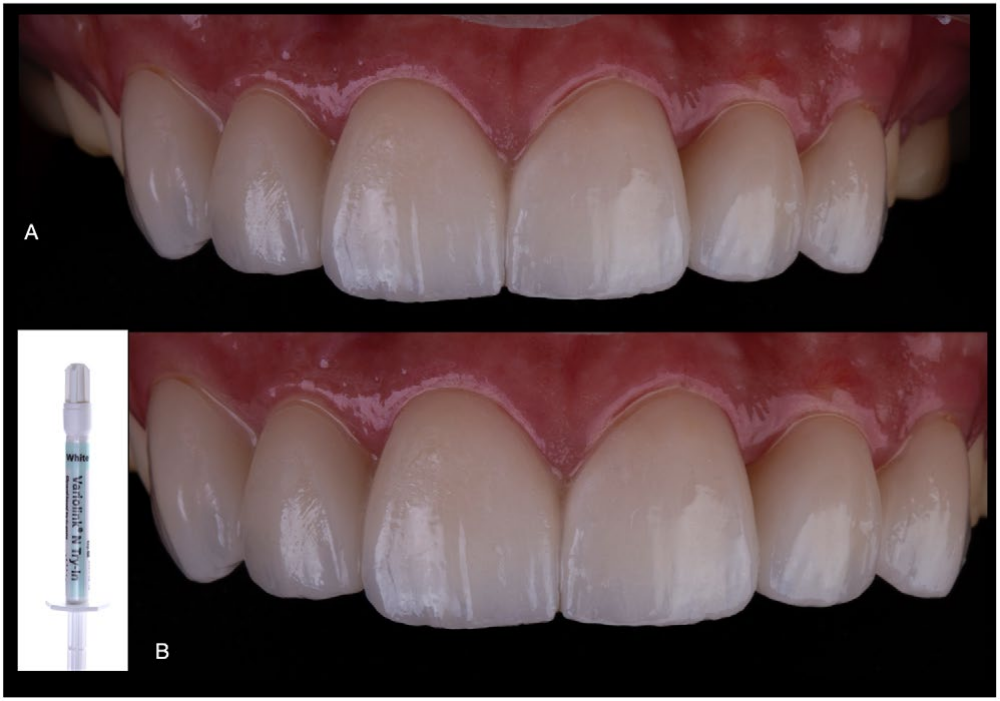
(A) Dry proof and (B) damp proof using try‐in paste (shade white, Variolink N, Ivoclar Vivadent) of lithium disilicate crowns in the anterior region.

Lithium–disilicate restorations were dry proof, the visual evaluation of the veneer on the tooth surface without any moisture or try‐in paste, to verify seating and interproximal contacts (Figure 12A). Subsequently, damp proof using try‐in paste (Figure 12B) was performed to select the ideal cement shade. The A1 shade of Variolink N (Ivoclar Vivadent, Liechtenstein) cement was selected, and preparations were cleaned with pumice and soft prophylactic brushes.

**FIGURE 13:**
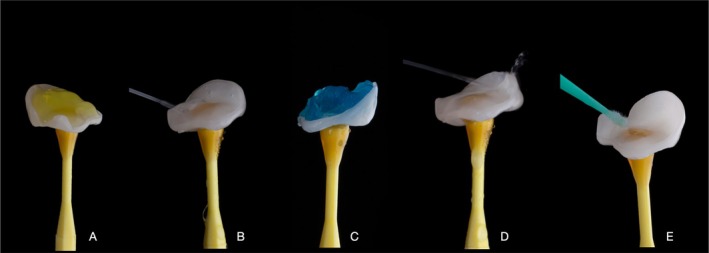
Partial bonded restorations conditioning protocol included (A) Hydroflorhidric acid etching (5%) for 20 s, (B) extensive water rinse, (C) cleaning with 37% orthophosphoric acid for 30 s, (D) water rinse and (E) silane application.

Lithium–disilicate restorations were cleaned in ethyl alcohol and subjected to the acid conditioning protocol recommended by the manufacturer. Hydrofluoric acid at 5% was applied to the inner surface of ceramic restorations for 20 s, washed for 40 s, and cleaned with 37% orthophosphoric acid for 30 s, followed by extensive water rinse. After drying, silane (Monobond, Ivoclar Vivadent, Liechtenstein) was applied and set to rest until solvent evaporation (Figure 13).

Tooth structure was conditioned according to the substrate. After relative isolation with Optragate (Ivoclar Vivadent, Liechtenstein), cotton rolls and polytetrafluoroethylene tape, selective acid etching (37% orthophosphoric acid) of the enamel was performed for 15 s, followed by water rinse, drying, and adhesive application (ClearFill SE bond, Kuraray, Japan). In dentin areas, ClearFill primer was applied, followed by adhesive application, as recommended by the manufacturer.

**FIGURE 14:**
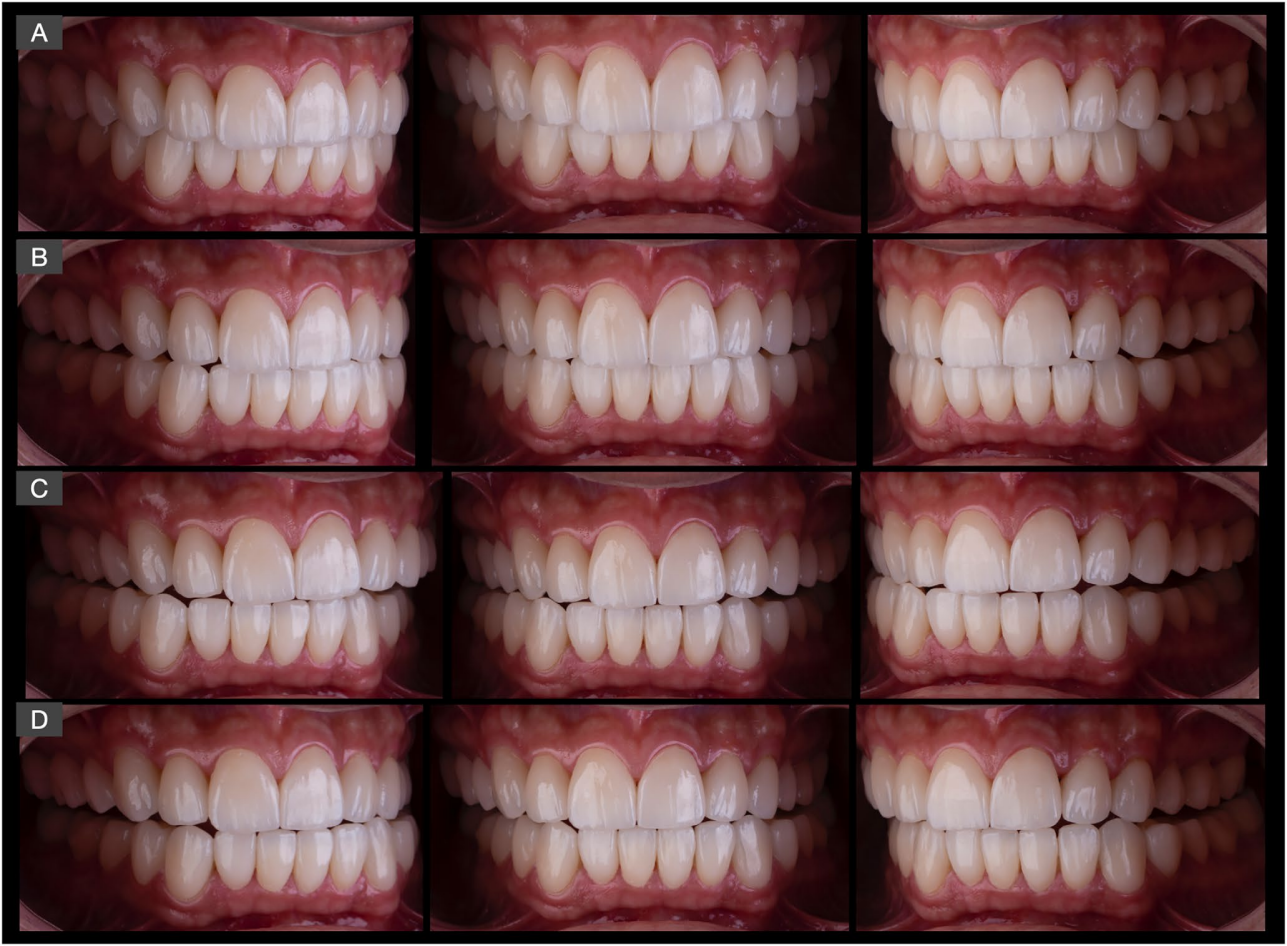
Frontal and lateral intraoral views after installation of all lithium disilicate bonded restorations and occlusal adjustments with evidence of the achievement of mutually protected occlusion during: (A) occlusion in centric relation, (B) mandibular protrusive movement, (C, D) lateral mandibular movements.

For cementation, a strategy of one‐by‐one element, from the anterior to the posterior region, was adopted. Resin cement (Variolink N base, white shade, Ivoclar Vivadent, Liechtenstein) was placed on the inner surface of the ceramic restoration, which was gently placed over the abutment until completed seating. Excess cement was removed with the aid of a brush and dental floss, and polymerization was performed with BluePhase PowerCure (Ivoclar Vivadent, Liechtenstein) with an intensity of 1200 mW/cm^2^ for 20 s on each surface, as recommended by the manufacturer. After cementation, excess cement was removed using a #12 blade (Swann‐Morton, England), and occlusal adjustments were performed to obtain simultaneous bilateral contacts in the posterior regions, as well as desocclusion provided by canines and incisors during lateral and protrusive movements, respectively (Figure 14A–D).

**FIGURE 15:**
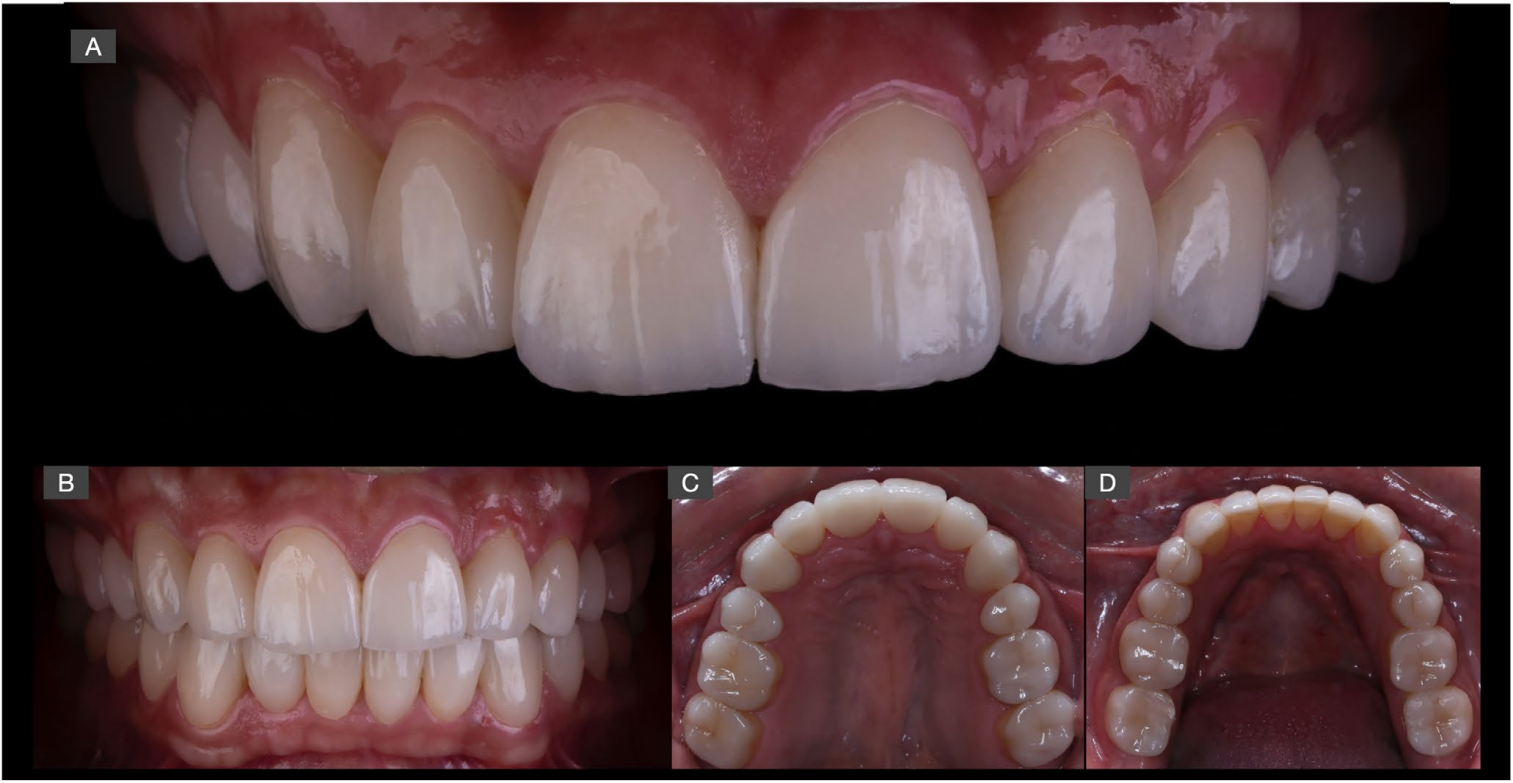
(A, B) Frontal views of the finalized treatment with a natural and esthetic appearance. (C, D) Occlusal views of maxillary and mandibular arches after installation and occlusal adjustments.

**FIGURE 16:**
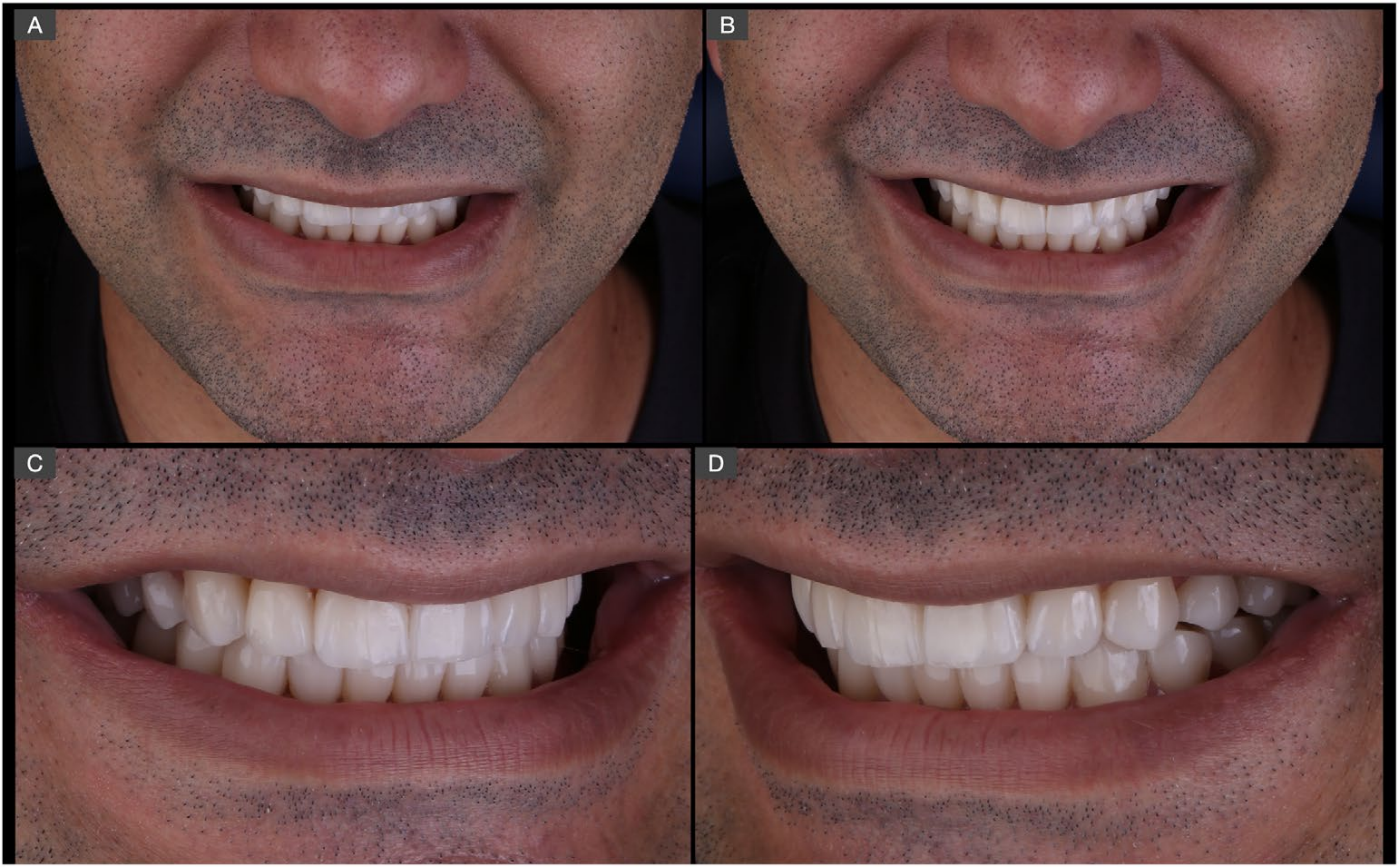
Final esthetic outcome of the complete oral rehabilitation with pressed lithium disilicate restorations at a close‐up frontal (A, B) and lateral (C, D) views of the smile.

The intraoral frontal and occlusal views of the finalized treatment, as well as the close‐up appearance of the oral rehabilitation are presented in Figures 15 and 16, respectively.

### 2.5 Three‐Dimensional Facial Evaluation Through Stereophotogrammetry

Stereophotogrammetry of the patient was performed at the initial condition, at mock‐up evaluation, and after finishing the treatment. Before obtaining the photos, anthropometric landmarks are pointed at the patient's face, and then the patient was instructed to keep his eyes open. The patient was seated with a head and neck support to maintain a natural, upright posture and to standardize head position across all imaging time points (pretreatment, mock‐up, and final). Then, the patient was asked to maintain the mandible closed at maximum intercuspation with his lips relaxed to assess VDO. The Vectra H1 portable stereophotogrammetry camera system (Canfield Scientific Inc., Fairfield, NJ, USA) was then used to take three consecutive pictures of the patient following the manufacturer's guidelines. Standardized floor markings were used to guide the execution of the photographic sequences. The photographic sequence consisted of three images; the first one was taken from a 45° angle on the patient's right side, with the camera positioned 20–30 cm below the face; the second from a direct frontal view; and the third from a 45° angle on the left side, maintaining the same vertical distance. Visual laser indicators (two converging green dots) ensured correct positioning by confirming the proper distance between the camera and the patient before each photograph was taken, as recommended by the manufacturer and reported in previous literature [54].

The three images captured were transferred to a computer and processed using the VAM Module Elaboration software, which automatically stitched them into a single 3D image. Each previously anthropometric landmark was digitally refined by zooming in and placing the marker precisely at the center of the designated location. Subsequently, linear, angular, and surface measurements of the lips were conducted [55].

**FIGURE 17:**
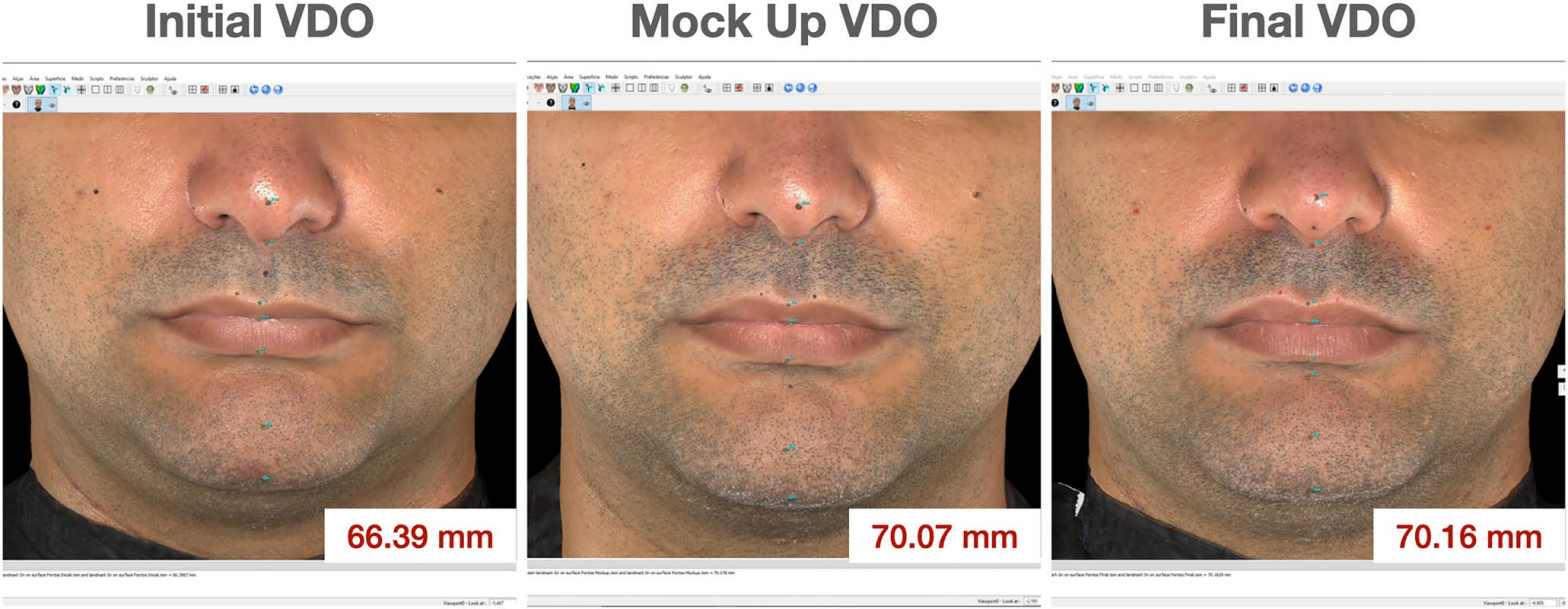
Vertical dimension of occlusion evaluation before treatment (initial VDO), at mock‐up stage, and after oral rehabilitation. An increase of approximately 4 mm is observed at the planning stage, and no significant differences are observed between the mock‐up evaluation and the outcome of the rehabilitation, which supports the predictability of the workflow utilized in this clinical case.

**FIGURE 18:**
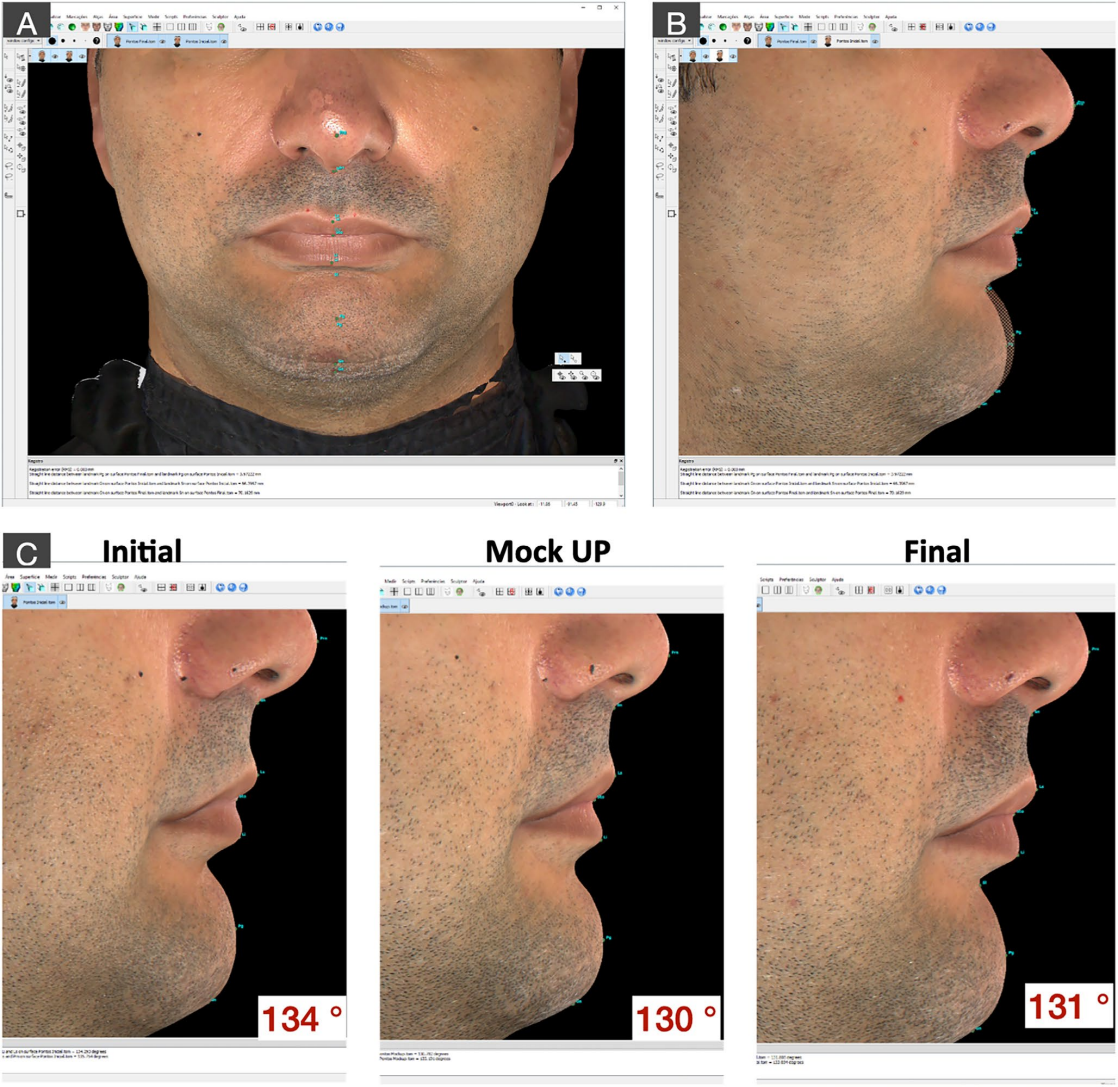
Overlapping of three‐dimensional stereophotogrammetry reconstructions obtained before and after treatment in the frontal (A) and lateral (B) views, where significant changes in the mandibular position due to the increase in vertical dimension of occlusion were observed. Additionally, a reduction of 3° in the nasolabial angle was observed after treatment (C).

Qualitative and quantitative evaluations were performed and demonstrated a significant impact of VDO rehabilitation on different esthetic parameters. 3D reconstruction demonstrated a gain of lip support as well as an increase in the VDO (Figures 17 and 18). Furthermore, a linear measurement of 66.39 mm was recorded for the VDO evaluation at baseline, and similar values were observed for the quantitative VDO evaluation between the mock‐up stage and the cemented final rehabilitation, with 70.07 mm and 70.16 mm, respectively (Figure 16).

Moreover, the overlapping of three‐dimensional stereophotogrammetry reconstructions (Figure 18) obtained before and after treatment depicted significant changes in the mandibular position due to the increase in the vertical dimension of occlusion, and a reduction of approximately 3° of the nasolabial angle, due to the achievement of proper lip support.

## 3 Discussion

The decrease in the VDO can be caused by parafunctional habits, such as bruxism, loss of posterior occlusal stability, congenital anomalies due to poor enamel formation, erosion caused by excessive ingestion of acidic substances or gastrointestinal disorders [2, 56]. Given the complexity of performing oral rehabilitation in patients with these characteristics, where all or most of the anatomic references for reconstruction have been lost, it is essential to determine a rehabilitation protocol that ensures the achievement of proper biological, functional, and esthetic outcomes predictably. The present clinical report presents a straightforward digital workflow to reconstruct patients with extensive tooth wear in need of reestablishment of the VDO. Notably, the application of 3D stereophotogrammetry demonstrated similar facial measurements for VDO assessment at the planning stage with an esthetic jig and at the end of the treatment, which demonstrates a high predictability of the treatment protocol.

Erosive tooth wear is a chemical–mechanical process that results in a cumulative loss of hard dental tissue [48]. Epidemiological data report a relatively high prevalence (~30%) [57] with increased wear over time [58]. Although erosive wear is multifactorial, acid exposure, whether extrinsic (diet) or intrinsic (reflux), is the primary cause [48, 59]. Early diagnosis and monitoring are essential to determine the need for preventive or restorative approaches. In this case, clinical records and photographs enabled tracking the patient's condition over 7 years, showing progression from moderate to severe wear through the basic erosive tooth wear evaluation. Additionally, a prior cosmetic treatment with resin composites in the anterior maxilla failed to address the patient's main concern. Dentin hypersensitivity, often a sign of progression, was present and required restorative intervention to relieve pain and restore esthetics and function. These restorative measures should be paired with preventive strategies and, when needed, medical referrals to manage underlying causes and prevent further damage [49].

Among several etiological factors, GERD, the effortless movement of stomach contents into the esophagus or mouth, has been strongly associated with dental erosion [39]. GERD has also been identified as an important risk factor for sleep bruxism, which in correlation with intrinsic acids, potentiates the loss of mineralized tooth structure [40]. The high prevalence of sleep bruxism observed in patients with GERD has led to the assumption that these conditions may share a common pathophysiological mechanism or etiological factor [39]. While the physiologic link between sleep bruxism, GERD, and increased salivation requires further investigation, it is essential to recognize that the association of chemical (reflux) and mechanical (bruxism) agents synergistically affects tooth structure [60] with a potential compromise of the VDO and facial esthetics [61]. Therefore, proper anamneses as well as facial and intraoral examination are crucial to determine the necessity of restorative intervention or the establishment of preventive and monitoring programs of care.

Although loss of tooth structure is diagnosed during the intra‐oral examination, VDO and VDR are both concepts based in facial analyses [3], thus intraoral evaluation itself should not be considered enough to determine if a patient has or has not a reduced VDO. Depending on the cause and the speed of dental wear, the loss of dental structure may be compensated with progressive tooth eruption accompanied or not by the basal bone [9, 10, 62]. In such scenario, a patient could present severe dental attrition or erosion and still maintain unaltered some facial esthetic parameters. Therefore, the determination of a new VDO should be performed considering facial esthetic and functional parameters and avoid arbitrary increase of the incisal pin of virtual or conventional semi‐adjustable articulator, as commonly made [1, 35]. Also, the Angle's classification determined when patients are in maximal intercuspal position should be considered when a new occlusal scheme is designed including CR and an increased VDO [63]. Additional tools have been recently brought to attention utilizing the “Smile Design and Space” concept [64]. This encompass a comprehensive pretreatment evaluation of the incisal edge position in relation to the face, the lip and smile dynamics, and the functional relationships between arches to ensure sufficient interoclusal space to increase VDO utilizing digital design [64]. Although previous literature had suggested that permanent increase of VDO of up to 5 mm is a safe and predictable procedure [29, 30], the evaluation of clinical parameters is imperative to reduce margins of error and the necessity of extensive occlusal and esthetic adjustments. While the utilization of long‐term provisional restorations as a “trial period” for increase VDO has been previously reported, there is no scientific evidence to validate a certain amount of time [28] neither to support the use of any appliance therapy before VDO alterations [65]. After mock‐up, phonetic and esthetic evaluations were performed in our patient and the planned maxillo‐mandibular position was maintained for the final restoration.

With the establishment of a new VDO and full‐arch rehabilitation, maximal intercuspal position could not serve as a reproducible starting point, requiring a new maxillo‐mandibular position independent of dental contacts [34]. Therefore, CR was adopted as a clinically useful, repeatable reference position to provide occlusal stability in the patient's occlusion during all phases of the prosthodontic treatment [34, 66]. Although recent literature has questioned the use of CR in favor of maximum intercuspation for dentate patients [32], full‐mouth rehabilitations still demand a reproducible mandibular reference [33]. Although efforts to identify the “correct” position of the temporomandibular joint condyle within the glenoid fossa are not supported and lack anatomical support, CR is useful as a maxillo‐mandibular reference position when extensive rehabilitation is needed and when the position of maximum intercuspation would be compromised during treatment [34]. Regardless of terminology preference, such as CR, reference position, or maxillo‐mandibular utility position [32, 33, 34, 35], the determination of a physiologic start‐point relation is key to allow for registration to be transferred to the dental technician.

In addition to establishing the patient's CR and VDO, esthetic parameters are essential to guide teeth proportions and position [67]. The incisal edge position of the maxillary central incisors, especially when combined with facial photographs, provides critical references for designing a full smile [68]. The determination of ideal incisor length and incisal plane regarding the lip at rest position and during smile has a fundamental role in complete oral rehabilitation planning [69] and can be assessed through different techniques, such as an esthetic jig [68, 70]. This chairside‐fabricated device offers a non‐invasive, cost‐effective, and time‐efficient approach to simultaneously assess both esthetic and functional parameters [70, 71].

Although several methods exist to determine the VDO, they all have limitations and should be used in association to reduce errors [67, 72]. The metric method, based on IOD and the subtraction of VDO from VDR, is among the most commonly used [4, 11]. Within this method, different devices can be employed, such as an L‐shaped bite gauge designed by Willis in 1930 [12]. Moreover, the same principle is used with similar devices such as the “jaw gauge,” or even with tongue depressors and reference points, which benefit from avoiding submental fat tissue as a support as is the case in the Willis bite gauge [73, 74]. Although metric and physiologic evaluations provide important information regarding the IOD, the association with esthetic and phonetic evaluations is highly encouraged due to the considerable variability in IOD among individuals [23].

The association of the esthetic jig, as a diagnostic tool, and the digital workflow presented in this case report allowed for a “test‐drive” stage performed through a full mouth mock‐up. This pivotal step allowed for the verification of the achievement of a tentative maxillo‐mandibular relation as well as the assessment of esthetic, phonetic, and occlusal parameters using a non‐invasive approach. After mock‐up approval by the patient and the verification of all esthetic and functional parameters, tooth preparation was guided by the maxillary and mandibular digital teeth arrangement [75, 76]. Tooth preparation aimed to provide enough space for the restorative material, respecting the minimum thickness required by the manufacturer. Moreover, in second maxillary and mandibular molars, a reduced IOD of approximately 1 mm was observed. To avoid extensive preparation in this region with potential compromise of pulp vitality, the authors decided to utilize ultra‐thin lithium disilicate partial crowns, with a minimum thickness of 0.5 mm. Although the long‐term behavior of ultra‐thin restorations has not been completely elucidated, some clinical trials have suggested high survival rates for bonded lithium disilicate restoration with occlusal thickness below 1 mm in one surface [37, 38]. Moreover, laboratorial studies have recommended caution for the indication of ultra‐thin restorations in areas of high mechanical loading [77].

The transfer of maxillo‐mandibular relationships is a critical step in oral rehabilitation, and it is not different in digital workflows. At the planning stage, all the references for the oral rehabilitation were determined in the esthetic jig. Therefore, a cautious tooth preparation sequence is required to allow for the transfer of that valuable information to another registration method that will maintain the VDO and CR record for intraoral scanning after tooth preparation. In this clinical case, the preparation of the anterior teeth which were not involved in the esthetic jig (maxillary and mandibular canines) allowed for the manufacture of registration copings over the maxillary canines with a stable contact against the prepared mandibular canines, which maintained the occlusal parameters determined by the esthetic jig. This simple procedure ensured the maintenance of all interocclusal relationships through the preparation and scanning process and minimal occlusal adjustments at the installation of the ceramic restorations. After cementation and occlusal adjustment and polishing, an occlusal splint was installed to protect the oral rehabilitation from the deleterious effects of sleep bruxism, and the patient was instructed on the importance of proper oral hygiene and frequent clinical follow‐ups.

Regarding the three‐dimensional evaluation of facial parameters, 3D stereophotogrammetry demonstrated high clinical applicability and was used in this clinical case to obtain volumetric and linear measurements to evaluate the VDO at the initial condition, during the mock‐up stage, and after treatment finalization. The overlapping of 3D images to detect facial and volumetric modifications allows identifying remarkably similar measurements of VDO at the mock‐up stage and after treatment finalization. These findings demonstrate the accuracy and predictability of the rehabilitation workflow utilized in this case, where the clinical parameters determined in the esthetic jig were successfully transferred to the mock‐up evaluation and from the prepared tooth to the virtual models and matched the result of the oral rehabilitation. It is undeniable that digital workflows simplify the rehabilitation process; however, the fundamental knowledge of oral rehabilitation and occlusal principles is paramount to planning the complete rehabilitation and establishing predictable protocols with the desired esthetic and functional outcomes. Stereophotogrammetry using the Vectra H1 camera and the Vectra Face Sculptor/VAM software may be an efficient method for quantitative assessment of the VDO in patients with extensive tooth wear, given that stereophotogrammetry has been considered the gold standard tool to evaluate facial soft tissue esthetics [78].

## 4 Conclusion

The proposed digital workflow allowed for a predictable rehabilitation of the VDO, where similar three‐dimensional measurements were observed during the mock‐up test and at the end of the treatment. 3D stereophotogrammetry imaging technology allowed for quantitatively assessing the differences in facial appearance after VDO rehabilitation.

## Conflicts of Interest

The authors declare no conflicts of interest.

## Data Availability

The data that support the findings of this study are available from the corresponding author upon reasonable request.

## References

[1] G. Goldstein , C. Goodacre , and K. MacGregor , “Occlusal Vertical Dimension: Best Evidence Consensus Statement,” Journal of Prosthodontics: Official Journal of the American College of Prosthodontists 30, no. S1 (2021): 12–19. 33783090 10.1111/jopr.13315

[2] D. R. Bloom and J. N. Padayachy , “Increasing Occlusal Vertical Dimension—Why, When and How,” British Dental Journal 200, no. 5 (2006): 251–256. 16528325 10.1038/sj.bdj.4813305

[3] GPT , “The Glossary of Prosthodontic Terms 2023: Tenth Edition,” Journal of Prosthetic Dentistry 130, no. 4 Suppl 1 (2023): e7–e126. 37914442 10.1016/j.prosdent.2023.03.002

[4] S. Sato , T. H. Hotta , and V. Pedrazzi , “Removable Occlusal Overlay Splint in the Management of Tooth Wear: A Clinical Report,” Journal of Prosthetic Dentistry 83, no. 4 (2000): 392–395. 10756287 10.1016/s0022-3913(00)70032-1

[5] W. C. Rivera‐Morales and N. D. Mohl , “Relationship of Occlusal Vertical Dimension to the Health of the Masticatory System,” Journal of Prosthetic Dentistry 65, no. 4 (1991): 547–553. 2066895 10.1016/0022-3913(91)90298-b

[6] R. Ajay , P. S. Manoharan , V. Rakshagan , B. M. OmarFarooq , P. Arunkumar , and R. Sasikala , “Correlation of Vertical Dimension of Occlusion in Parents and Their Offspring: A Cephalometric Study,” Journal of Pharmacy & Bioallied Sciences 11, no. Suppl 2 (2019): S371–S375. 31198371 10.4103/JPBS.JPBS_37_19 PMC6555371

[7] B. P. LeSage , “CAD/CAM: Applications for Transitional Bonding to Restore Occlusal Vertical Dimension,” Journal of Esthetic and Restorative Dentistry 32, no. 2 (2020): 132–140. 31823502 10.1111/jerd.12554 PMC7328720

[8] Y. Kaifu , “Tooth Wear and Compensatory Modification of the Anterior Dentoalveolar Complex in Humans,” American Journal of Physical Anthropology 111, no. 3 (2000): 369–392. 10685038 10.1002/(SICI)1096-8644(200003)111:3<369::AID-AJPA6>3.0.CO;2-#

[9] A. Margvelashvili , C. P. Zollikofer , D. Lordkipanidze , T. Peltomaki , and M. S. Ponce de Leon , “Tooth Wear and Dentoalveolar Remodeling Are Key Factors of Morphological Variation in the Dmanisi Mandibles,” Proceedings of the National Academy of Sciences of the United States of America 110, no. 43 (2013): 17278–17283. 24101504 10.1073/pnas.1316052110 PMC3808665

[10] B. G. Levers and A. I. Darling , “Continuous Eruption of Some Adult Human Teeth of Ancient Populations,” Archives of Oral Biology 28, no. 5 (1983): 401–408. 6354154 10.1016/0003-9969(83)90135-8

[11] M. A. Pleasure , “Correct Vertical Dimension and Freeway Space,” Journal of the American Dental Association 43, no. 2 (1951): 160–163. 14850211 10.14219/jada.archive.1951.0188

[12] F. Willis , “Esthetics of Full Denture Construction,” Journal of the American Dental Association 17, no. 4 (1922): 636–642.

[13] G. F. McGee , “Use of Facial Measurements in Determining Vertical Dimension,” Journal of the American Dental Association 35, no. 5 (1947): 342–350. 20257138 10.14219/jada.archive.1947.0361

[14] S. Subhas , D. Singh , A. Gupta , M. Kesari , A. Kumar , and L. Nayak , “Facial Measurements: A Guide for Vertical Dimension,” Journal of Family Medicine and Primary Care 9, no. 4 (2020): 2056. 10.4103/jfmpc.jfmpc_968_19 PMC7346898 32670965

[15] M. Niswonger , “The Rest Position of the Mandible and the Centric Relation,” Journal of the American Dental Association 21, no. 9 (1922): 1572–1582.

[16] M. M. Silverman , “Accurate Measurement of Vertical Dimension by Phonetics and the Speaking Centric Space. Part I,” Dental Digest 57, no. 6 (1951): 261–265. 14840049

[17] M. M. Silverman , “Accurate Measurement of Vertical Dimension by Phonetics and the Speaking Centric Space: Part Two,” Dental Digest 57, no. 7 (1951): 308–311. 14849219

[18] A. N. Ryakhovsky , D. N. Dedkov , R. S. Gvetadze , and E. A. Boytsova , “Cephalometric Estimation of Vertical Dimension of Occlusion,” Stomatologiia 96, no. 1 (2017): 63–71. 10.17116/stomat201796163-71 36475399

[19] J. E. Pyott and A. Schaeffer , “Centric Relation and Vertical Dimension by Cephalometric Roentgenograms,” Journal of Prosthetic Dentistry 4, no. 1 (1954): 35–41.

[20] J. G. Gattozzi , B. R. Nicol , G. W. Somes , and C. W. Ellinger , “Variations in Mandibular Rest Positions With and Without Dentures in Place,” Journal of Prosthetic Dentistry 36, no. 2 (1976): 159–163. 1068279 10.1016/0022-3913(76)90137-2

[21] E. Pound , “Controlling Anomalies of Vertical Dimension and Speech,” Journal of Prosthetic Dentistry 36, no. 2 (1976): 124–135. 789862 10.1016/0022-3913(76)90134-7

[22] F. Fayz , A. Eslami , and G. N. Graser , “Use of Anterior Teeth Measurements in Determining Occlusal Vertical Dimension,” Journal of Prosthetic Dentistry 58, no. 3 (1987): 317–322. 3476738 10.1016/0022-3913(87)90048-5

[23] R. P. Harper , “Clinical Indications for Altering Vertical Dimension of Occlusion. Functional and Biologic Considerations for Reconstruction of the Dental Occlusion,” Quintessence International 31, no. 4 (2000): 275–280. 11203936

[24] R. Chapman and N. Mehta , “The Craniofacial Complex and Vertical Dimension: Proper Positioning Improves Speech, Breathing, Eating and Appearance,” Inside Dentistry 9 (2013): 54–78.

[25] W. C. Rivera‐Morales and N. D. Mohl , “Restoration of the Vertical Dimension of Occlusion in the Severely Worn Dentition,” Dental Clinics of North America 36, no. 3 (1992): 651–664. 1397430

[26] C. H. Schuyler , “Problems Associated With Opening the Bite Which Would Contraindicate It as a Common Procedure,” Journal of the American Dental Association 26, no. 5 (1939): 734–740.

[27] R. W. Tench , “Dangers in Dental Reconstruction In‐Volving Increase of the Vertical Dimension of the Lower Third of the Human Face,” Journal of the American Dental Association and the Dental Cosmos 25, no. 4 (1938): 566–570.

[28] L. Lassmann , M. A. Calamita , and D. Manfredini , “Myths Surrounding Vertical Dimension of Occlusion in Restorative Dentistry: A Scoping Review,” Journal of Esthetic and Restorative Dentistry: Official Publication of the American Academy of Esthetic Dentistry 37 (2024): 94–105. 10.1111/jerd.13303 39189329

[29] I. Moreno‐Hay and J. P. Okeson , “Does Altering the Occlusal Vertical Dimension Produce Temporomandibular Disorders? A Literature Review,” Journal of Oral Rehabilitation 42, no. 11 (2015): 875–882. 26140528 10.1111/joor.12326

[30] J. Abduo , “Safety of Increasing Vertical Dimension of Occlusion: A Systematic Review,” Quintessence International 43, no. 5 (2012): 369–380. 22536588

[31] G. Fabbri , R. Sorrentino , G. Cannistraro , et al., “Increasing the Vertical Dimension of Occlusion: A Multicenter Retrospective Clinical Comparative Study on 100 Patients With Fixed Tooth‐Supported, Mixed, and Implant‐Supported Full‐Arch Rehabilitations,” International Journal of Periodontics & Restorative Dentistry 38, no. 3 (2018): 323–335. 29641621 10.11607/prd.3295

[32] A. J. J. Zonnenberg , J. C. Turp , and C. S. Greene , “Centric Relation Critically Revisited‐What Are the Clinical Implications?,” Journal of Oral Rehabilitation 48, no. 9 (2021): 1050–1055. 34164832 10.1111/joor.13215

[33] C. Fornai , I. Tester , K. Parlett , C. Basili , and H. N. Costa , “Centric Relation: A Matter of Form and Substance,” Journal of Oral Rehabilitation 49, no. 7 (2022): 687–690. 35377510 10.1111/joor.13329 PMC9324986

[34] D. Manfredini , C. Ercoli , C. E. Poggio , F. Carboncini , and M. Ferrari , “Centric Relation‐A Biological Perspective of a Technical Concept,” Journal of Oral Rehabilitation 50, no. 11 (2023): 1355–1361. 37394665 10.1111/joor.13553

[35] G. Goldstein and J. P. Wiens , “The Occlusion Project, Evidence for Its Future,” Journal of Prosthodontics: Official Journal of the American College of Prosthodontists 30, no. S1 (2021): 3–4. 33783086 10.1111/jopr.13311

[36] E. A. Tsitrou , M. Helvatjoglu‐Antoniades , and R. van Noort , “A Preliminary Evaluation of the Structural Integrity and Fracture Mode of Minimally Prepared Resin Bonded CAD/CAM Crowns,” Journal of Dentistry 38, no. 1 (2010): 16–22. 19683378 10.1016/j.jdent.2009.07.003

[37] K. A. Malament , M. Margvelashvili‐Malament , Z. S. Natto , V. Thompson , D. Rekow , and W. Att , “10.9‐Year Survival of Pressed Acid Etched Monolithic e.Max Lithium Disilicate Glass‐Ceramic Partial Coverage Restorations: Performance and Outcomes as a Function of Tooth Position, Age, Sex, and the Type of Partial Coverage Restoration (Inlay or Onlay),” Journal of Prosthetic Dentistry 126 (2020): 523–532. 33012530 10.1016/j.prosdent.2020.07.015

[38] K. A. Malament , M. Margvelashvili‐Malament , Z. S. Natto , V. Thompson , D. Rekow , and W. Att , “Comparison of 16.9‐Year Survival of Pressed Acid Etched e.Max Lithium Disilicate Glass‐Ceramic Complete and Partial Coverage Restorations in Posterior Teeth: Performance and Outcomes as a Function of Tooth Position, Age, Sex, and Thickness of Ceramic Material,” Journal of Prosthetic Dentistry 126, no. 4 (2021): 533–545. 33010922 10.1016/j.prosdent.2020.08.013

[39] C. M. Mengatto , C. S. Dalberto , B. Scheeren , and S. G. Barros , “Association Between Sleep Bruxism and Gastroesophageal Reflux Disease,” Journal of Prosthetic Dentistry 110, no. 5 (2013): 349–355. 24011800 10.1016/j.prosdent.2013.05.002

[40] A. Nota , L. Pittari , M. Paggi , S. Abati , and S. Tecco , “Correlation Between Bruxism and Gastroesophageal Reflux Disorder and Their Effects on Tooth Wear. A Systematic Review,” Journal of Clinical Medicine 11, no. 4 (2022): 1107. 35207380 10.3390/jcm11041107 PMC8879082

[41] C. Sforza , M. de Menezes , and V. Ferrario , “Soft‐ and Hard‐Tissue Facial Anthropometry in Three Dimensions: What's New,” Journal of Anthropological Sciences 91 (2013): 159–184. 23833019 10.4436/jass.91007

[42] Z. H. Feng , Y. Dong , S. Z. Bai , et al., “Virtual Transplantation in Designing a Facial Prosthesis for Extensive Maxillofacial Defects That Cross the Facial Midline Using Computer‐Assisted Technology,” International Journal of Prosthodontics 23, no. 6 (2010): 513–520. 21209985

[43] C. L. Heike , K. Upson , E. Stuhaug , and S. M. Weinberg , “3D Digital Stereophotogrammetry: A Practical Guide to Facial Image Acquisition,” Head & Face Medicine 6 (2010): 18. 20667081 10.1186/1746-160X-6-18 PMC2920242

[44] C. Runte , D. Dirksen , H. Delere , et al., “Optical Data Acquisition for Computer‐Assisted Design of Facial Prostheses,” International Journal of Prosthodontics 15, no. 2 (2002): 129–132. 11951801

[45] M. G. R. Pucciarelli , G. H. L. Toyoshima , K. H. Neppelenbroek , et al., “Evaluation of the Labial Protrusion and Lip Esthetic Changes After Complete Denture Treatment Through Stereophotogrammetry,” International Journal of Prosthodontics 37, no. 6 (2023): 1–25. 10.11607/ijp.8623 37988431

[46] M. G. R. Pucciarelli , H. de Lima , G. Toyoshima , T. M. de Oliveira , K. H. Neppelenbroek , and S. Soares , “Quantifying the Facial Proportions in Edentulous Individuals Before and After Rehabilitation With Complete Dentures Compared With Dentate Individuals: A 3D Stereophotogrammetry Study,” Journal of Prosthetic Dentistry 131, no. 4 (2024): 697–704. 35431030 10.1016/j.prosdent.2022.03.013

[47] J. F. Cardoso , M. G. R. Pucciarelli , J. A. S. Laurenti , et al., “Arch Symmetry in Patients Without and With Cleft Lip and Palate After Orthodontic/Rehabilitative Treatment—A Stereophotogrammetry Study,” Cleft Palate‐Craniofacial Journal 60, no. 12 (2023): 1565–1571. 10.1177/10556656221110096 35769043

[48] T. S. Carvalho , P. Colon , C. Ganss , et al., “Consensus Report of the European Federation of Conservative Dentistry: Erosive Tooth Wear—Diagnosis and Management,” Clinical Oral Investigations 19, no. 7 (2015): 1557–1561. 26121968 10.1007/s00784-015-1511-7

[49] D. Bartlett , C. Ganss , and A. Lussi , “Basic Erosive Wear Examination (BEWE): A New Scoring System for Scientific and Clinical Needs,” Clinical Oral Investigations 12, no. Suppl 1 (2008): S65–S68. 18228057 10.1007/s00784-007-0181-5 PMC2238785

[50] S. M. Parsel , E. L. Wu , C. A. Riley , and E. D. McCoul , “Gastroesophageal and Laryngopharyngeal Reflux Associated With Laryngeal Malignancy: A Systematic Review and Meta‐Analysis,” Clinical Gastroenterology and Hepatology 17, no. 7 (2019): 1253–1264.e5. 30366155 10.1016/j.cgh.2018.10.028

[51] C. A. Riley , M. J. Marino , M. C. Hsieh , E. L. Wu , X. C. Wu , and E. D. McCoul , “Detection of Laryngeal Carcinoma in the U.S. Elderly Population With Gastroesophageal Reflux Disease,” Head and Neck 41, no. 5 (2019): 1434–1440. 30681216 10.1002/hed.25600

[52] A. C. Eells , C. Mackintosh , L. Marks , and M. J. Marino , “Gastroesophageal Reflux Disease and Head and Neck Cancers: A Systematic Review and Meta‐Analysis,” American Journal of Otolaryngology 41, no. 6 (2020): 102653. 32841763 10.1016/j.amjoto.2020.102653

[53] M. A. Akl , D. E. Mansour , K. Mays , and A. G. Wee , “Mathematical Tooth Proportions: A Systematic Review,” Journal of Prosthodontics: Official Journal of the American College of Prosthodontists 31, no. 4 (2022): 289–298. 34463403 10.1111/jopr.13420

[54] D. Gibelli , V. Pucciarelli , A. Cappella , C. Dolci , and C. Sforza , “Are Portable Stereophotogrammetric Devices Reliable in Facial Imaging? A Validation Study of VECTRA H1 Device,” Journal of Oral and Maxillofacial Surgery 76, no. 8 (2018): 1772–1784. 29458028 10.1016/j.joms.2018.01.021

[55] K. S. Fioravanti , M. G. R. Mengoa , L. V. Paludetto , et al., “Comparative Analysis of Lip Morphology in Brazilian Caucasian Individuals Between 20 and 50 Years Old Using Stereophotogrammetry,” Oral and Maxillofacial Surgery 29, no. 1 (2025): 57. 39964586 10.1007/s10006-025-01349-z

[56] A. C. Pavarina , A. L. Machado , C. E. Vergani , and E. T. Giampaolo , “Overlay Removable Partial Dentures for a Patient With Ectodermal Dysplasia: A Clinical Report,” Journal of Prosthetic Dentistry 86, no. 6 (2001): 574–577. 11753305 10.1067/mpr.2001.119981

[57] D. W. Bartlett , A. Lussi , N. X. West , P. Bouchard , M. Sanz , and D. Bourgeois , “Prevalence of Tooth Wear on Buccal and Lingual Surfaces and Possible Risk Factors in Young European Adults,” Journal of Dentistry 41, no. 11 (2013): 1007–1013. 24004965 10.1016/j.jdent.2013.08.018

[58] H. C. Eto , F. Miranda , D. Rios , et al., “Erosive Tooth Wear in Subjects With Normal Occlusion: A Pioneering Longitudinal Study up to the Age of 60,” Journal of Clinical Medicine 12, no. 19 (2023): 6318. 37834962 10.3390/jcm12196318 PMC10573230

[59] A. Lussi and T. S. Carvalho , “Erosive Tooth Wear: A Multifactorial Condition of Growing Concern and Increasing Knowledge,” Monographs in Oral Science 25 (2014): 1–15. 24993253 10.1159/000360380

[60] P. C. R. Conti , C. O. Cunha , A. Conti , L. R. Bonjardim , J. S. Barbosa , and Y. M. Costa , “Secondary Bruxism: A Valid Diagnosis or Just a Coincidental Finding of Additional Masticatory Muscle Activity? A Narrative Review of Literature,” Journal of Oral Rehabilitation 51, no. 1 (2024): 74–86. 37688286 10.1111/joor.13592

[61] C. Schirra , “Loss of Vertical Dimension: Extensive Therapy in Dentitions With Erosion and Abrasion. A Treatment Strategy for the Dental Practitioner,” Quintessence International 44, no. 10 (2013): 733. 24078974 10.3290/j.qi.a30181

[62] D. C. Berry and D. F. Poole , “Attrition: Possible Mechanisms of Compensation,” Journal of Oral Rehabilitation 3, no. 3 (1976): 201–206. 1068232 10.1111/j.1365-2842.1976.tb00945.x

[63] S. Campbell and G. Goldstein , “Angle's Classification‐A Prosthodontic Consideration: Best Evidence Consensus Statement,” Journal of Prosthodontics: Official Journal of the American College of Prosthodontists 30, no. S1 (2021): 67–71. 33331655 10.1111/jopr.13307

[64] L. Lassmann , M. A. Calamita , and M. B. Blatz , “The ‘Smile Design and Space’ Concept for Altering Vertical Dimension of Occlusion and Esthetic Restorative Material Selection,” Journal of Esthetic and Restorative Dentistry: Official Publication of the American Academy of Esthetic Dentistry 37 (2024): 56–67. 10.1111/jerd.13317 39295223

[65] L. Crins , N. J. M. Opdam , C. M. Kreulen , E. M. Bronkhorst , M. Huysmans , and B. A. C. Loomans , “Randomised Controlled Trial on Testing an Increased Vertical Dimension of Occlusion Prior to Restorative Treatment of Tooth Wear,” Journal of Oral Rehabilitation 50, no. 4 (2023): 267–275. 36582043 10.1111/joor.13408

[66] C. Millet , C. Jeannin , B. Vincent , and G. Malquarti , “Report on the Determination of Occlusal Vertical Dimension and Centric Relation Using Swallowing in Edentulous Patients,” Journal of Oral Rehabilitation 30, no. 11 (2003): 1118–1122. 14641678 10.1046/j.1365-2842.2003.01201.x

[67] M. Calamita , C. Coachman , N. Sesma , and J. Kois , “Occlusal Vertical Dimension: Treatment Planning Decisions and Management Considerations,” International Journal of Esthetic Dentistry 14, no. 2 (2019): 166–181. 31061997

[68] S. Finkel and P. Pizzi , “Dentist‐Ceramist Communication: Protocols for an Effective Esthetic Team,” Dental Clinics of North America 64, no. 4 (2020): 697–708. 32888517 10.1016/j.cden.2020.06.005

[69] F. Spear , “The Maxillary Central Incisal Edge: A Key to Esthetic and Functional Treatment Planning,” Compendium of Continuing Education in Dentistry 20, no. 6 (1999): 512–516. 10650364

[70] G. Romeo , G. Iliev , and G. Gürel , “Central Incisor Mock‐Up: A Fundamental Role in Esthetic Rehabilitation,” Quintessence of Dental Technology 45 (2023): 116.

[71] A. Flavio , “THE ESTHETIC JIG: An Original Dental Appliance for Esthetic Rehabilitation of Occlusal Vertical Dimension,” Journal of Cosmetic Dentistry 34, no. 4 (2019): 46.

[72] J. D. Rugh and C. J. Drago , “Vertical Dimension: A Study of Clinical Rest Position and Jaw Muscle Activity,” Journal of Prosthetic Dentistry 45, no. 6 (1981): 670–675. 6941018 10.1016/0022-3913(81)90426-1

[73] M. N. Alhajj , N. Khalifa , J. Abduo , A. G. Amran , and I. A. Ismail , “Determination of Occlusal Vertical Dimension for Complete Dentures Patients: An Updated Review,” Journal of Oral Rehabilitation 44, no. 11 (2017): 896–907. 28600914 10.1111/joor.12522

[74] M. Morikawa , Y. Kozono , B. S. Noguchi , and S. Toyoda , “Reproducibility of the Vertical Dimension of Occlusion With an Improved Measuring Gauge,” Journal of Prosthetic Dentistry 60, no. 1 (1988): 58–61. 3042984 10.1016/0022-3913(88)90352-6

[75] S. Koubi , G. Gurel , P. Margossian , R. Massihi , and H. Tassery , “A Simplified Approach for Restoration of Worn Dentition Using the Full Mock‐Up Concept: Clinical Case Reports,” International Journal of Periodontics and Restorative Dentistry 38, no. 2 (2018): 189–197. 29447311 10.11607/prd.3186

[76] G. Gurel , “Porcelain Laminate Veneers: Minimal Tooth Preparation by Design,” Dental Clinics of North America 51, no. 2 (2007): 419–431. 17532920 10.1016/j.cden.2007.03.007

[77] E. B. Benalcazar Jalkh , I. S. Ramalho , E. T. P. Bergamo , et al., “Ultrathin Lithium Disilicate and Translucent Zirconia Crowns for Posterior Teeth: Survival and Failure Modes,” Journal of Esthetic and Restorative Dentistry: Official Publication of the American Academy of Esthetic Dentistry 36, no. 2 (2024): 381–390. 10.1111/jerd.13127 37676053

[78] A. Rossetti , M. De Menezes , R. Rosati , V. F. Ferrario , and C. Sforza , “The Role of the Golden Proportion in the Evaluation of Facial Esthetics,” Angle Orthodontist 83, no. 5 (2013): 801–808. 23477386 10.2319/111812-883.1 PMC8744514

